# Switching Ionization Polarity to Simplify MS/MS Sequencing of Digital Polymers: the Case of Informational Poly(Amino phosphodiester)s

**DOI:** 10.1002/rcm.70047

**Published:** 2026-02-01

**Authors:** Isaure Sergent, Ian Roszak, Jean‐François Lutz, Laurence Charles

**Affiliations:** ^1^ Aix Marseille Université, CNRS, Institut de Chimie Radicalaire (ICR) Marseille France; ^2^ Université de Strasbourg, CNRS, Institut de Science et d'Ingénierie Supramoléculaires (ISIS) Strasbourg France

**Keywords:** digital polymers, informational polymers, ionization polarity, MS/MS sequencing, poly(amino phosphodiester)s

## Abstract

**Rationale:**

MS/MS sequencing is commonly used to read binary messages encoded in digital polymers. To achieve full sequence coverage required for error‐free reading, the structure of coding units is usually optimized to prevent extensive signal dilution over multiple fragmentation routes. Changing ionization polarity can also have a significant effect on MS/MS pattern, which is explored here for poly(amino phosphodiester)s.

**Methods:**

Poly(amino phosphodiester)s (N‐PPDEs) include comonomers composed of one phosphate group and a main‐chain tertiary amine holding different alkyl substituents as coding moieties. Accordingly, they can be readily ionized in both polarity modes, using ammonium acetate to promote electrospray formation of deprotonated species and protonated molecules when switching from negative to positive ion mode. Changes of their MS/MS spectra are studied with regard to the behavior of their PPDE homologues lacking the main‐chain tertiary amine.

**Results:**

In collision‐induced dissociation (CID) conditions, eight fragment series are produced upon cleavage of all phosphate bonds in deprotonated species, whereas only four ion series are generated from protonated N‐PPDEs in which O–P–O linkages remain intact. The advantageous MS/MS behavior of protonated N‐PPDEs has been rationalized by considering that protons located on tertiary amines are also solvated by nearby phosphate groups, promoting exclusive cleavage of C–O bonds.

**Conclusions:**

In contrast to PPDEs, switching from deprotonated to protonated precursors yields highly simplified CID data for N‐PPDEs, opening promising perspectives for reliable MS/MS sequencing of long coded polymers.

## 1 Introduction

Over the last 10 years, digital polymers have emerged as new media for information storage. These macromolecules have a uniform chain length and exhibit a controlled sequence of monomers [1]. However, in contrast to biomolecules whose primary structure is dictated by biological functions, the sequence of digital polymers is purposely defined to write messages [2, 3]. Their repeating units are indeed considered as letters of an alphabet, the most popular and simple one consisting of two comonomers respectively assigned to the 0‐ and 1‐bit of the ASCII code. Storage density can easily be increased with a larger set of comonomers, for example, 2 bits per monomer when using four units defined as 00, 01, 10, and 11, respectively [4, 5]. Reading such molecularly encoded information consists of determining the polymer sequence: As long as coding monomers have different masses, tandem mass spectrometry (MS/MS) is to date the most efficient sequencing methodology to decode these messages [6]. The principle of MS/MS sequencing is based on the production of series of fragments that are obtained after the cleavage of the same backbone bonds in all repeating units. As a result, each series contains fragments that still hold one or the other chain termination and differ in mass by a single building unit. Their mass analysis then allows the entire primary structure to be reconstructed, either from a single complete fragment series or combination thereof.

As for any synthetic polymer subjected to MS/MS, the number and type of fragment series of digital polymers depend on their backbone chemistry [7]. Yet, the main purpose of informational polymers is to be read and one mandatory condition to accurately decipher coded messages is to achieve full sequence coverage. So, early in their development [8], it has been acknowledged that the structure of their repeating units can be tailored to minimize the variety of dissociation reactions. Indeed, the occurrence of multiple fragmentation pathways jeopardizes full sequence coverage. On the one hand, this strongly contributes to signal dilution over various fragment series, which abundance rapidly decreases as the chain length increases, hence compromising their detection. On the other hand, this mainly increases redundancy of sequencing information, hence providing marginal added value for sequence reconstruction. Accordingly, controlling the dissociation behavior of digital chains is key to maintain full sequence coverage of digital polymers as their length increases. When using collision‐induced dissociation (CID), one efficient strategy to minimize the number of dissociation routes consists of combining chemical groups with bonds of very different dissociation energies: Fine‐tuning of activation level then allows cleavage of the weakest linkages while leaving any other bonds intact. This was shown by Zhang and co‐workers with the marked decrease of C–S bond dissociation energy upon oxidation of thioether into sulfoxide moieties [9], whereas our group achieved full control of coded chain fragmentation with weak alkoxyamine bonds distributed between blocks of phosphodiester monomers [10].

**SCHEME 1:**
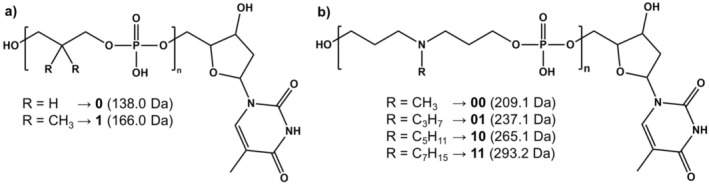
General structure of (a) PPDEs and (b) N‐PPDEs and nomenclature of monomers as a function of their R substituent. The terminal thymidine comes from the solid support.

Besides this couple of examples, it should be acknowledged that the number of fragment series is barely predictable. Accordingly, one key rule guiding the design of any new digital polymers is to incorporate chemical groups that promote efficient ionization so that production of abundant precursor ions guarantees detectability of sequencing fragments in case of multiple series. Yet, a compromise has often to be made between ideal monomer structure and constraints related to synthesis, which has to be efficient at producing long and uniform chains. In this context, the most promising digital polymers developed in our group are undoubtedly sequence‐defined poly(phosphodiester)s (PPDEs). These polymers are synthesized by solid‐phase iterative phosphoramidite chemistry (PPC), a technique initially developed for the chemical synthesis of DNA [11], which can, however, be used with a variety of nonnatural building blocks [12, 13]. Moreover, this synthesis approach can be automated when implemented on a DNA synthesizer, which permits production of long uniform chains [14], as in our initial work [15] where PPDEs with more than 100 repeating units were prepared using an alphabet based on two symbols (Scheme 1a). PPDEs contain one phosphate group per repeating unit and readily deprotonate in the negative ion mode electrospray ionization (ESI). In CID conditions, deprotonated PPDEs typically dissociate via efficient cleavage of all phosphate bonds, leading to four sets of complementary fragments [16]. This leads to rich MS/MS data but sequence information is highly redundant: Full sequence coverage is readily achieved for PPDEs with less than 50 repeating units, but for chains above this size, a large number of fragments provides information on the same pieces of sequence, whereas other parts remain uncovered. Changing ionization polarity does not solve this issue: Not only are protonated PPDEs produced with low yield in positive mode ESI but their CID still generates eight fragments series, as previously shown for oligonucleotides when studied in the negative [17] or in the positive [18] ion mode.

In great contrast to PPDEs, the recently developed poly(amino phosphodiester)s family (N‐PPDEs) [19] offers a very different scenario. Also produced by automated PPC, these digital polymers are made of repeating units in which the coding R moieties are no longer connected to a carbon atom but to a nitrogen atom (Scheme 1b). While deprotonated chains exhibit the same dissociation behavior as PPDEs, CID of protonated molecules proceeds via cleavage of two (instead of four) bonds in each phosphate group. Accordingly, this greatly reduces signal dilution in MS/MS while maintaining coverage of the whole sequence. Using a set of different oligomers (Table 1), we have investigated mechanisms underlying these dissociation reactions to rationalize the peculiar behavior of protonated N‐PPDEs, which has also enabled us to revisit fragment ion assignments performed in our previous study [19].

**TABLE 1:** Binary sequence and mass of N‐PPDEs with 00 (209.1 Da), 01 (237.1 Da), 10 (265.1 Da), and 11 (293.2 Da).

	Sequence	Mass, Da
P1	01‐00‐01‐00‐01‐00‐01‐00	2026.8
P2	00‐01‐01‐01‐00‐00‐00‐00	1998.9
P3	01‐00‐01‐01‐00‐01‐01‐01	2082.9
P4	10‐11‐11‐01‐10‐00‐10‐01	2307.2
P5	11‐11‐10‐00‐01‐10‐10‐11	2363.3
P6	00‐01‐10‐11‐00‐01‐10‐11	2251.1
P7	00‐01‐10‐11‐11‐10‐01‐00	2251.1
P8	00‐01‐01‐01‐01‐00‐00‐00‐00‐00‐00‐01‐01‐01‐01‐00	3811.5

## 2 Experimental Section

### 2.1 Chemicals

Acetonitrile, water, and methanol used to prepare polymer samples were from VWR International (Fontenay‐sous‐Bois, France). Ammonium acetate used as an ionizing agent was purchased from Sigma‐Aldrich (St Louis, MO).

### 2.2 Digital N‐PPDE Oligomers

All N‐PPDE oligomers investigated herein were synthesized by automated PPC on controlled pore glass solid support [15]. As detailed in a recent study [19] and shown in Scheme 1b, a set of four comonomers was employed in which the alkyl side‐chain connected to the main‐chain nitrogen atom is of increasing size: methyl for 00, propyl for 01, pentyl for 10, and heptyl for 11. Details of the eight oligomers used in this study are provided in Table 1.

### 2.3 Mass Spectrometry

All MS and MS/MS experiments were performed with a ZenoTOF mass spectrometer (Sciex, Concord, Ontario, Canada). Dry samples were dissolved in 300 μL H_2_O/ACN (50/50, v/v), further diluted (1/10 to 1/100) in a methanolic solution of ammonium acetate (3 mM), and introduced in the ESI source with a syringe pump at a 10 μL min^−1^ flow rate. The ESI source was operated under a nebulizing gas (air, 20 psi) and heated at 35°C using the following settings: negative mode, capillary voltage: −4.8 kV, cone voltage: −75 V; positive mode, capillary voltage: +5.5 kV, cone voltage: +75 V. In this hybrid instrument, ions were measured using an orthogonal acceleration time‐of‐flight (oa‐TOF) mass analyzer. In the MS/MS mode, a quadrupole was used for selection of precursor ions to be further submitted to CID in a collision cell filled with N_2_. Accurate mass measurements were performed using internal calibration. Instrument control, data acquisition, and data processing of all experiments were achieved using the Sciex OS software (3.4.0) provided by Sciex.

## 3 Results and Discussion

### 3.1 Electrospray Ionization of N‐PPDEs

**FIGURE 1:**
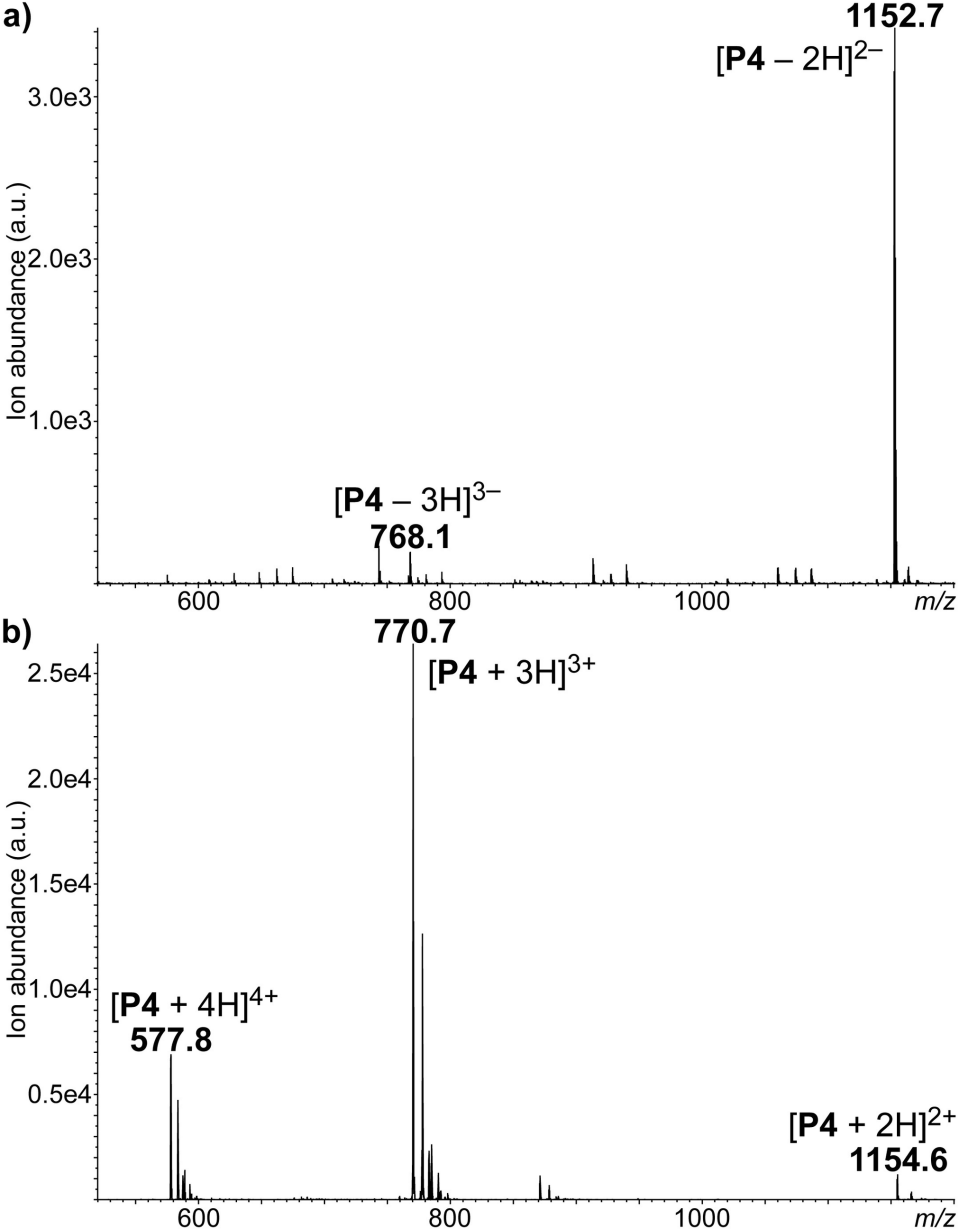
ESI mass spectra recorded for the P4 oligomer (a) in the negative ion mode and (b) in the positive ion mode.

Owing to the chemical structure of their monomers containing one phosphate group and one tertiary amine (Scheme 1b), N‐PPDEs can ionize in both polarity modes when subjected to ESI. On the one hand, phosphate groups readily deprotonate in the negative ion mode and, as previously observed for PPDEs [16], the preferential charge state roughly corresponds to one deprotonated phosphate group out of three. This is exemplified in Figure 1a with the P4 octamer observed with a major abundance at the 2− charge state (*m/z* 1152.7), whereas the intensity of [P4—3H]^3−^ ion at *m/z* 768.1 is more than 15 times lower. On the other hand, the presence of one tertiary amine group in each repeating unit highly favors protonation in positive mode ESI. As observed in Figure 1b, P4 is distributed over three different charge states but appears to be more stable in the gas phase as [P4 + 3H]^3+^ at *m/z* 770.7. Nevertheless, comparing ion absolute abundance clearly indicates that, in contrast to PPDEs lacking the tertiary amine, N‐PPDEs exhibit a much higher ionization yield in the positive than in the negative mode ESI. This is best shown in Figure S1 where the intensity of [P4 + 3H]^3+^ is nearly eight times higher than that of [P4 − 2H]^2−^. Accordingly, production of high amounts of precursor ions in the positive ion mode will guarantee best detectability of their CID fragments.

### 3.2 CID of Deprotonated N‐PPDEs

**FIGURE 2:**
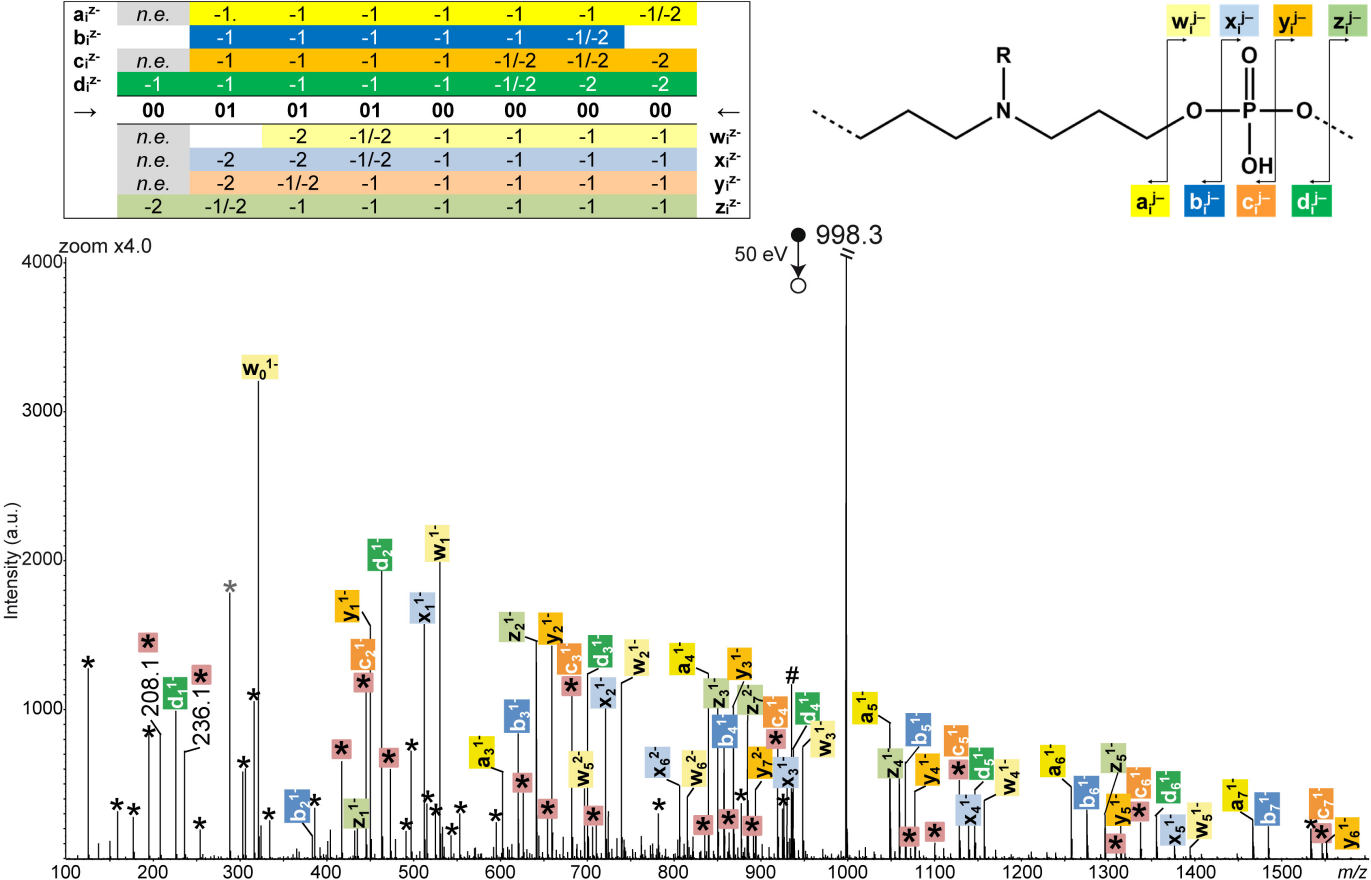
MS/MS spectrum of [P2 − 2H]^2−^ at *m/z* 998.3, with fragmentation scheme showing nomenclature of fragments (top right) and sequence coverage table of P2 (top left) indicating the charge state(s) of detected fragments (*n.e*.: not expected). Asterisks designate secondary fragments, and those inserted in pink squares are for internal fragments with *m/z* = ∑*m* (Mi) − 1. Number sign designates the doubly charged fragment at *m/z* 935.4 formed after loss of the terminal T base.

In the negative ion mode, CID of deprotonated N‐PPDEs proceeds according to the same reactions previously observed for PPDEs [16], yielding eight product ion series. This is exemplified in Figure 2 with the P2 octamer at the 2− charge state. In this CID spectrum, fragments are named after the nomenclature established by Wesdemiotis et al. for polymeric fragments [7], as shown in the fragmentation scheme of Figure 2.

**SCHEME 2:**
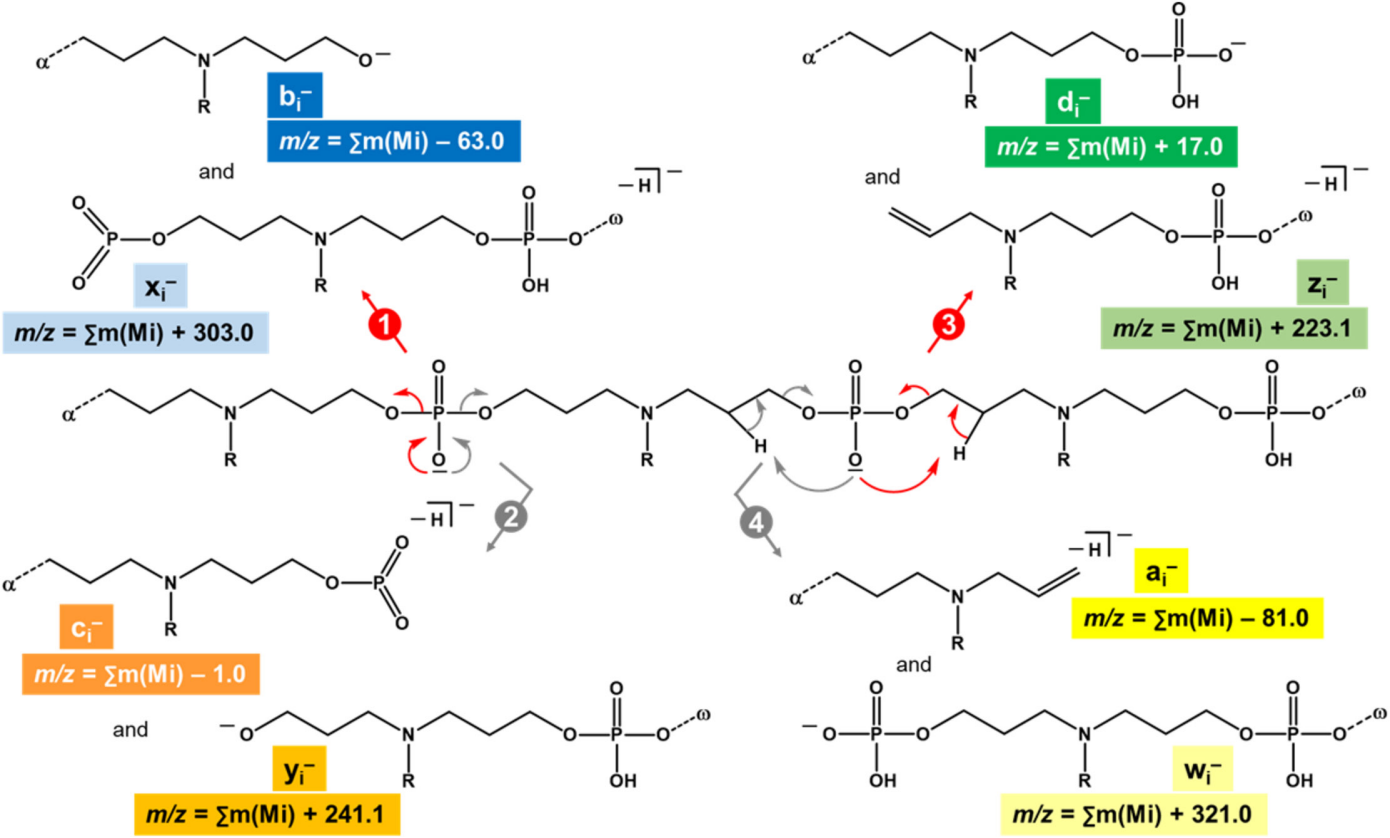
Proposed mechanisms for phosphate bond cleavages in doubly deprotonated N‐PPDEs, yielding fragment series b_i_^−^ and x_i_^−^ (pathway 1, in red), c_i_^−^ and y_i_^−^ (pathway 2, in grey), d_i_^−^ and z_i_^−^ (pathway 3, in red), and a_i_^−^ and w_i_^−^ (pathway 4, in grey). In equations defining *m/z* values for each series, *m* (Mi) corresponds to the mass of comonomers: *m*(00) = 209.1 Da, *m*(01) = 237.1 Da, *m*(10) = 265.1 Da, and *m*(11) = 293.2 Da.

Charge‐assisted mechanisms proposed in Scheme 2 for the formation of the four sets of complementary fragments are inspired by dissociation reactions described for small oligonucleotides [17]. Detection of a doubly charged fragment at *m/z* 935.4 (designated by # in Figure 2) reveals loss of the thymine base (126.0 Da) from the ω termination. Apart from this fragment, because their repeating units contain neither sugar nor base moieties, deprotonated N‐PPDEs exclusively dissociate via cleavage of phosphate bonds. Although the largest product ions are also detected as doubly charged species (see sequence coverage table in Figure 2), activation of doubly deprotonated precursors permits us to envisage pairs of complementary singly charged fragments to be obtained for each of the four pathways shown in Scheme 2. Structures proposed for all ion series are supported by accurate mass measurements of fragments detected in the CID spectrum of [P2 − 2H]^2−^ (Tables S1–S4). All fragments series are nearly complete and mainly lack those members that cannot be formed (“not expected” in the sequence coverage table of Figure 2). For example, considering the structure proposed for a_i_^−^ ions in Scheme 2, the smallest member of this series needs to contain two monomers to be detected as a deprotonated species (so a_1_^−^ is not expected).

Overall, de novo sequencing can be performed from any of these eight fragment series but using the c_i_^−^ ion series alone to reconstruct the sequence is not convenient. Indeed, amongst secondary fragments that do no longer contain any of the original chain terminations (i.e., internal fragments), some were found to share the same elemental composition as c_i_^−^ ions. In Figure 2, they are designated by asterisks in pink squares. The structure of these internal fragments is composed of entire monomers only (Table S5) so, similar to c_i_^−^ ions, their *m/z* value is defined as *m/z* = ∑m (Mi) − 1.0. The smallest ions verifying this equation are at *m/z* 208.1 and *m/z* 236.1: Because c_1_^−^ cannot exist (Scheme 2), these two species can safely be identified as internal fragments, namely, deprotonated units [00 − H]^−^ and [01 − H]^−^, respectively. However, as their size increases, members of this internal fragment series do not allow unequivocal assignment of c_i_^−^ ions. For example, the CID spectrum of [P2 − 2H]^2−^ exhibits three ions that all could be assigned to one c_2_^−^ fragment depending on its sequence: *m/z* 417.2 for α‐00‐00, *m/z* 445.2 for α‐00‐01 or α‐01‐00, and *m/z* 473.2 for α‐01‐01. Only the lack of complementary fragments for both *m/z* 417.2 and *m/z* 473.2 permits to correctly assign c_2_^−^ at *m/z* 445.2 formed complementarily to y_6_^−^ at *m/z* 1551.6. The same issue is found for higher congeners of this series (Table S5). In practice, for de novo sequencing, c_i_^−^ ions are assigned once the primary structure of N‐PPDEs has been established from other MS/MS fragment series.

### 3.3 CID of Protonated N‐PPDEs

**FIGURE 3:**
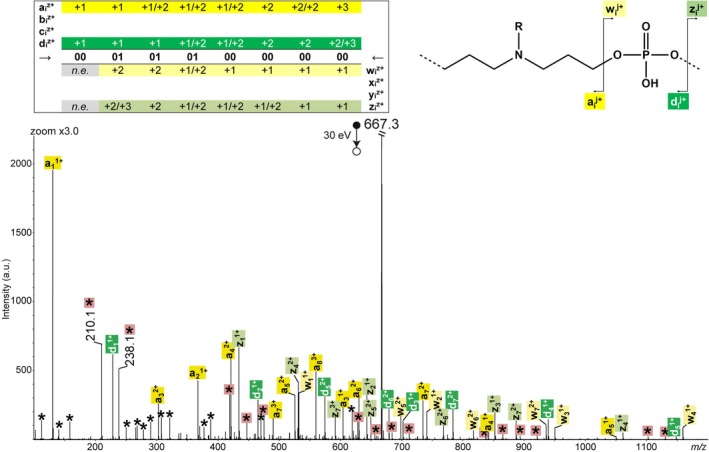
MS/MS spectrum of [P2 + 3H]^3+^ at *m/z* 667.3, with fragmentation scheme showing nomenclature of fragments (top right) and sequence coverage table of P2 (top left), indicating the charge state(s) of detected fragments (*n.e*.: not expected). Asterisks designate secondary fragments, and those inserted in pink squares are for internal fragments with *m/z* = ∑*m* (Mi) + 1.

**FIGURE 4:**
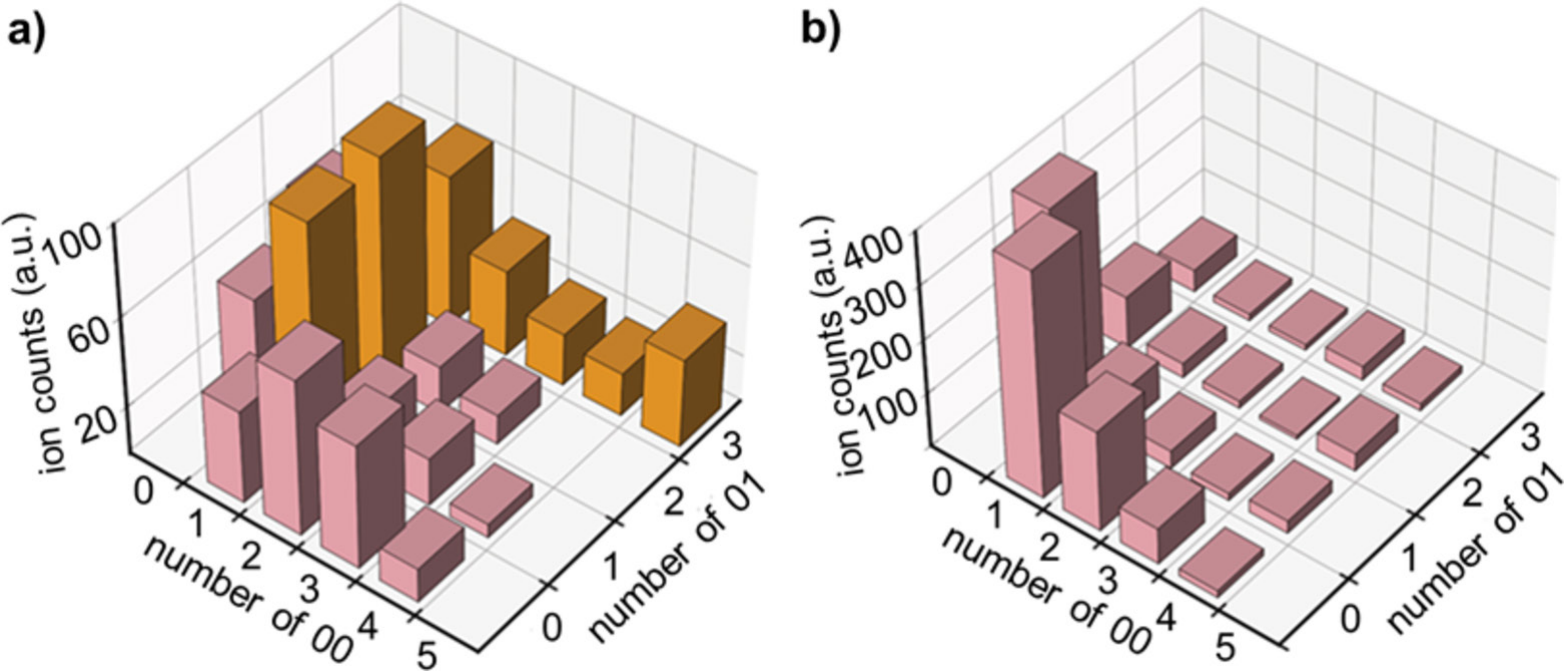
Abundance of P2 fragments measured at (a) *m/z* = ∑m (Mi) − 1 in the negative ion mode and (b) *m/z* = ∑m (Mi) + 1 in the positive ion mode, as a function of their comonomeric composition. Internal fragments are in pink, while those also corresponding to c_i_ fragments are in orange.

Collisional activation of protonated N‐PPDEs leads to ESI(+)‐MS/MS spectra that are far less complex than CID data recorded in the negative ion mode. Instead of the eight main fragment series generated from [P2 − 2H]^2−^ (Figure 2), [P2 + 3H]^3+^ is observed to dissociate into only four series of sequencing fragments (Figure 3), namely, a_i_^j+^/w_i_^j+^ and d_i_^j+^/z_i_^j+^. As illustrated by the dissociation scheme shown in Figure 3, these pairs of complementary ions are formed after cleavage of C–O or O–C bonds in phosphodiester moieties, while there is no evidence for cleavage of O–P–O bonds. Additional fragments observed in this CID spectrum correspond to internal fragments (designated by asterisks in Figure 3), including series composed of entire units only with *m/z* = ∑m (Mi) + 1 (designated by asterisks in pink squares in Figure 3). As previously discussed for MS/MS data recorded in the negative mode, some of these ions might have been assigned to the c_i_^+^ fragments of [P2 + 3H]^3+^ (e.g., c_2_^+^ at *m/z* 447.2, c_3_^+^ at *m/z* 684.3, c_4_^+^ at *m/z* 921.4, c_5_^+^ at *m/z* 1130.5, …) formed upon cleavage of P–O bonds. These were actually the assignments made in our previous publication [19]. However, these assignments have been ruled out for two main reasons. First, complementary y_i_^+^ fragments expected upon cleavage of P–O bonds are never detected. Second, putative c_i_^+^ fragments do not exhibit the same intensity pattern compared with c_i_^−^ fragments in the negative mode. When plotting the abundance of all ions with *m/z* = ∑m (Mi) − 1 detected in the negative mode CID spectrum of [P2 − 2H]^2−^ (Figure 4a), those actually corresponding to c_i_^−^ fragments (in orange) have a major contribution. In contrast, a very different pattern is observed when plotting the abundance of all ions with *m/z* = ∑m (Mi) + 1 detected in the positive mode CID spectrum of [P2 + 3H]^3+^ (Figure 4b): Major species are protonated monomers, and signal abundance remains quite low for ions detected at those *m/z* values expected for c_i_^+^ fragments, suggesting that all are actually internal fragments.

**SCHEME 3:**
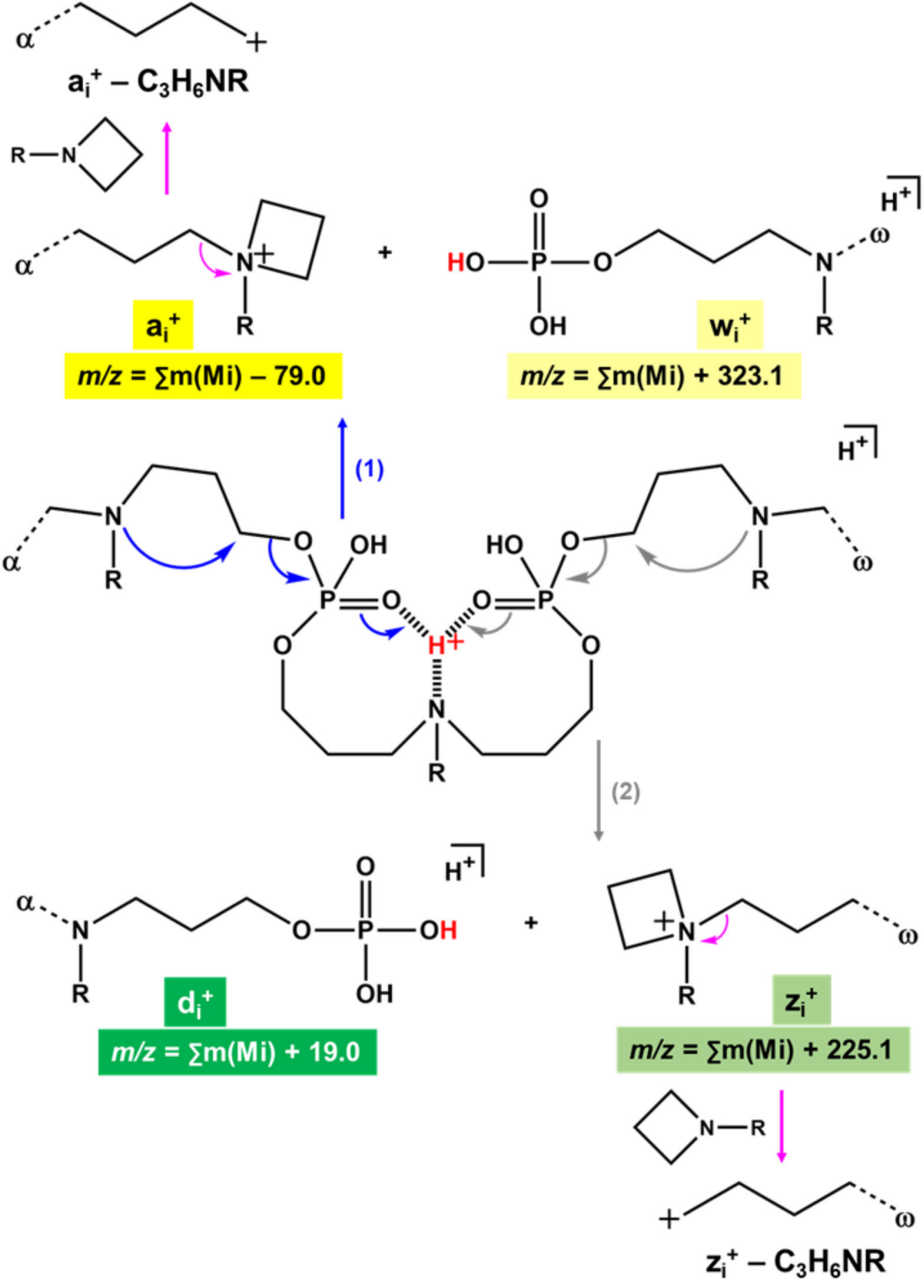
Proposed mechanisms for dissociation of multiply protonated N‐PPDEs, yielding fragment series a_i_^j+^ and w_i_^j+^ (pathway 1, in blue) and d_i_^j+^ and z_i_^j+^ (pathway 2, in grey). Subsequent loss of the four‐membered ring moiety as a C_3_H_6_NR neutral from both a_i_^j+^ and z_i_^j+^ ions is depicted with pink arrows.

In order to rationalize the lack of O–P–O bond cleavage, the structure shown in Scheme 3 has been proposed for protonated precursors. This structure assumes preferential location of adducted protons on tertiary amines of N‐PPDE repeating units. This is supported by the fact that loss of neutral thymine (126.0 Da) remains very minor (the so‐formed triply charged *m/z* 625.3 fragment has an abundance of 30 a.u. in Figure 3), consistent with the low proton affinity of thymine [20]. This structure also shows that, once adducted to the tertiary amine, the proton (in red) is also solvated by the two nearby P=O groups. Accordingly, opening of one or the other P=O double bonds to create a new PO–H linkage would always induce cleavage of one O–C bond (but neither O–P nor P–O). Then, depending on the location of the dissociating phosphate group being prior to or after the protonated tertiary amine, similar mechanisms would proceed to produce pairs of complementary fragments: a_i_^j+^/w_i_^j+^ fragments according to pathway 1 (in blue, Scheme 3) and d_i_^j+^/z_i_^j+^ fragments according to pathway 2 (in grey, Scheme 3). Elemental composition of all fragments is supported by accurate mass measurements (Tables S6–S7). Owing to the proposed pathways, all fragments are expected in both a_i_^j+^ and d_i_^j+^ series, whereas the largest member of w_i_^j+^ and z_i_^j+^ series cannot form. Formation of the charged nitrogen‐containing four‐membered ring in both a_i_^j+^ and z_i_^j+^ ions is supported by subsequent elimination of C_3_H_6_NR from these two types of fragments (pink arrows in Scheme 3), as observed during pseudo‐MS^3^ experiments. Mass of the released C_3_H_6_NR neutral depends on the R group initially connected to the charged nitrogen, and thus on the last unit of the dissociating ion. For example, a_2_^+^ of sequence α‐00‐01 is observed to eliminate C_3_H_6_N–C_3_H_7_ (99.1 Da) whereas C_3_H_6_N–CH_3_ (71.1 Da) is lost from z_2_^+^ of sequence ω‐00‐00 (Figure S2).

**FIGURE 5:**
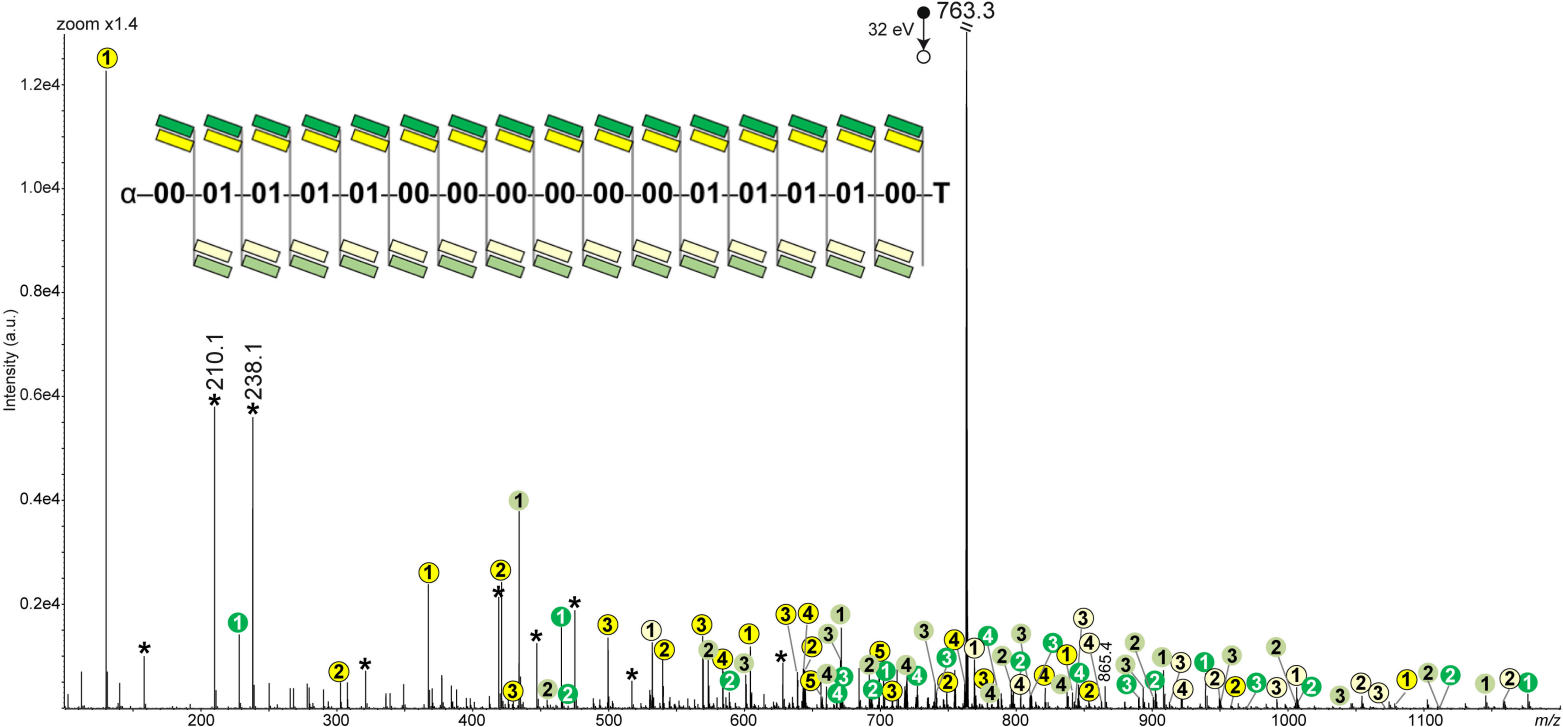
MS/MS spectrum of [P8 + 5H]^5+^ at *m/z* 763.3, with sequence coverage shown in inset. For the sake of clarity, fragments are not annotated with their symbols but by circles of different colors (a_i_^z+^, yellow; d_i_^z+^, green; w_i_^z+^, pale yellow; z_i_^z+^, pale green) that include their charge state. Asterisks designate secondary fragments.

Similar CID patterns with only four series of sequencing fragments are obtained for all tested octamers (Figures S3–S8), including those with monomers containing long R segments, that is, R = C_5_H_11_ in coding unit 10 and R = C_7_H_15_ in coding unit 11. This shows that bulky repeating units do not modify the dissociation behavior of protonated N‐PPDEs. In all cases, full sequence reconstruction is achieved with no error from any of the four complete fragment series. This is notably exemplified with MS/MS data recorded for isobaric octamers P6 and P7 that share the same comonomeric composition but different sequences, namely, 00‐01‐10‐11‐00‐01‐10‐11 and 00‐01‐10‐11‐11‐10‐01‐00, respectively (Figures S7–S8). In addition, increasing the chain length (and so the charge state of precursor ions) does not modify the fragmentation pattern observed for protonated N‐PPDEs. This is documented in Figure 5 for [P8 + 5H]^5+^ at *m/z* 763.3, where the sequence of 16 repeating units is readily recovered with no error from any of the four fragment series (Tables S8–S9).

## 4 Conclusion

Peculiar changes of the MS/MS behavior of N‐PPDEs when switching from the negative to the positive ion mode could be rationalized. Notably, comparing CID data recorded for deprotonated and protonated oligomers has permitted us to solve the ambiguity between some internal fragments and c ions. This detailed analysis allows us to safely conclude that dissociation of protonated N‐PPDEs only proceeds via two main pathways, yielding two pairs of complementary product ion series. Accordingly, selecting the positive ion mode to perform MS/MS sequencing offers major benefits to achieve the full sequence coverage requested for error‐free reading of information stored in digital N‐PPDEs. First, protonated N‐PPDEs dissociate into four main fragment series, which is half as many as their deprotonated counterparts: This efficiently minimizes signal dilution in CID spectra, which is key to detect all product ions. Second, this simple dissociation behavior is observed to proceed independently of the length of the R coding moiety (from CH_3_ in 00 units to C_7_H_15_ in 11 units). Because the main‐chain nitrogen atom in N‐PPDEs is a convenient site for side‐chain functionalization, a larger set of comonomers spanning a wider variety of R coding groups can be readily envisaged to further increase storage density. Finally, N‐PPDEs are produced with much higher yield as protonated species compared with deprotonated molecules. Selecting highly abundant precursor ions in CID is an additional advantage to detect all their fragments, the number of which rapidly increases with the number of coding units. Altogether, this opens promising perspectives for increasing the amount of readable information in a single chain, which is a usual bottleneck in the development of digital polymers.

### Associated Content

Ion abundance in both ESI polarities; accurate mass measurements of fragments of [P2 − 2H]^2−^; structure and *m/z* values of internal fragments; accurate mass measurements of fragments of [P2 + 3H]^3+^; pseudo‐MS^3^ of a_2_^+^ and z_2_^+^ fragments from [P2 + 3H]^3+^; CID spectra of [P1 + 3H]^3+^, [P3 + 3H]^3+^, [P4 + 3H]^3+^, [P5 + 3H]^3+^, [P6 + 3H]^3+^, and [P7 + 3H]^3+^; accurate mass measurements of fragments of [P8 + 5H]^5+^.

## Author Contributions

**Isaure Sergent:** investigation. **Ian Roszak:** investigation. **Jean‐François Lutz:** writing – review and editing, resources, supervision. **Laurence Charles:** writing – original draft, conceptualization, validation, resources, supervision, writing – review and editing.

## Funding

This study was supported by the Agence Nationale de la Recherche, ANR‐19‐CE06‐0020‐01, ANR‐19‐CE06‐0020‐02.

## Conflicts of Interest

The authors declare no conflicts of interest.

## Supporting information


**Figure S1:** Abundance of P4 ions as a function of ESI polarity.
**Table S1:** Accurate mass measurement of a_i_^j−^ and w_i_^j−^ fragments of [P2 − 2H]^2−^.
**Table S2:** Accurate mass measurement of b_i_^j−^ and x_i_^j−^ fragments of [P2 − 2H]^2−^.
**Table S3:** Accurate mass measurement of c_i_^j−^ and y_i_^j−^ fragments of [P2 − 2H]^2−^.
**Table S4:** Accurate mass measurement of d_i_^j−^ and z_i_^j−^ fragments of [P2 − 2H]^2−^.
**Table S5:** Internal fragments with *m/z* = ∑m(Mi) + 1.
**Table S6:** Accurate mass measurement of a_i_^j+^ and w_i_^j+^ fragments of [P2 + 3H]^3+^.
**Table S7:** Accurate mass measurement of d_i_^j+^ and z_i_^j+^ fragments of [P2 + 3H]^3+^.
**Figure S2:** Pseudo‐MS^3^ of a_2_^+^ and z_2_^+^ fragments of [P2 + 3H]^3+^.
**Figure S3:** MS/MS of [P1 + 3H]^3+^ at *m/z* 676.6.
**Figure S4:** MS/MS of [P3 + 3H]^3+^ at *m/z* 695.3.
**Figure S5:** MS/MS of [P4 + 3H]^3+^ at *m/z* 770.1.
**Figure S6:** MS/MS of [P5 + 3H]^3+^ at *m/z* 788.8.
**Figure S7:** MS/MS of [P6 + 3H]^3+^ at *m/z* 751.4.
**Figure S8:** MS/MS of [P7 + 3H]^3+^ at *m/z* 751.4.
**Table S8:** Accurate mass measurement of a_i_^j+^ and w_i_^j+^ fragments of [P8 + 5H]^5+^.
**Table S9:** Accurate mass measurement of d_i_^j+^ and z_i_^j+^ fragments of [P8 + 5H]^5+^.

## Data Availability

The data that support the findings of this study are available from the corresponding authors upon reasonable request.
