# Supplementary material for: Switching Ionization Polarity to Simplify MS/MS Sequencing of Digital Polymers: the Case of Informational Poly(Amino phosphodiester)s
Source: Rapid Commun Mass Spectrom. 2026 Feb 1;40(9):e70047. doi: 10.1002/rcm.70047 (PMC12861711; doi:10.1002/rcm.70047)
Supplement: Supplementary file 1 — Figure S1: Abundance of P4 ions as a function of ESI polarity. Table S1: Accurate mass measurement of ai j− and wi j− fragments of [P2 − 2H]2−. Table S2: Accurate mass measurement of bi j− and xi j− fragments of [P2 − 2H]2−. Table S3: Accurate mass measurement of ci j− and yi j− fragments of [P2 − 2H]2−. Table S4: Accurate mass measurement of di j− and zi j− fragments of [P2 − 2H]2−. Table S5: Internal fragments with m/z = ∑m(Mi) + 1. Table S6: Accurate mass measurement of ai j+ and wi j+ fragments of [P2 + 3H]3+. Table S7: Accurate mass measurement of di j+ and zi j+ fragments of [P2 + 3H]3+. Figure S2: Pseudo‐MS3 of a2 + and z2 + fragments of [P2 + 3H]3+. Figure S3: MS/MS of [P1 + 3H]3+ at m/z 676.6. Figure S4: MS/MS of [P3 + 3H]3+ at m/z 695.3. Figure S5: MS/MS of [P4 + 3H]3+ at m/z 770.1. Figure S6: MS/MS of [P5 + 3H]3+ at m/z 788.8. Figure S7: MS/MS of [P6 + 3H]3+ at m/z 751.4. Figure S8: MS/MS of [P7 + 3H]3+ at m/z 751.4. Table S8: Accurate mass measurement of ai j+ and wi j+ fragments of [P8 + 5H]5+. Table S9: Accurate mass measurement of di j+ and zi j+ fragments of [P8 + 5H]5+. [file RCM-40-e70047-s001.docx]

**Switching ionization polarity to simplify MS/MS sequencing of digital polymers: the case of informational poly(aminophosphodiester)s**

Isaure Sergent,^1^ Ian Roszak,^2^ Jean-François Lutz,^2*^ Laurence Charles^1^*

^1^ Aix Marseille Université, CNRS, Institut de Chimie Radicalaire (ICR), 13397 Marseille Cedex 20, France

^2^ Université de Strasbourg, CNRS, Institut de Science et d’Ingénierie Supramoléculaires (ISIS), 67000 Strasbourg, France

*Corresponding authors:

Prof. Laurence Charles, Aix Marseille Université, CNRS, UMR 7273, Institut de Chimie Radicalaire, 13397 Marseille Cedex 20, France. E-mail: [laurence.charles@univ-amu.fr](mailto:laurence.charles@univ-amu.fr)

Dr Jean-François Lutz, Université de Strasbourg, CNRS, Institut de Science et d’Ingénierie Supramoléculaires (ISIS), 67000 Strasbourg, France. E-mail: [jflutz@unistra.fr](mailto:jflutz@unistra.fr)

| Table of content | page |
| --- | --- |
| **Figure S1.** Abundance of P4 ions as a function of ESI polarity ……………………….. | S2 |
| **Table S1.** Accurate mass measurement of a_i_^j–^ and w_i_^j–^ fragments of [P2 – 2H]^2–^ ……... | S3 |
| **Table S2.** Accurate mass measurement of b_i_^j–^ and x_i_^j–^ fragments of [P2 – 2H]^2–^ ……… | S3 |
| **Table S3.** Accurate mass measurement of c_i_^j–^ and y_i_^j–^ fragments of [P2 – 2H]^2–^ ……… | S4 |
| **Table S4.** Accurate mass measurement of d_i_^j–^ and z_i_^j–^ fragments of [P2 – 2H]^2–^ ……… | S4 |
| **Table S5.** Internal fragments with *m/z* = ∑m(Mi) + 1 …………………………………. | S5 |
| **Table S6.** Accurate mass measurement of a_i_^j+^ and w_i_^j+^ fragments of [P2 + 3H]^3+^ …….. | S6 |
| **Table S7.** Accurate mass measurement of d_i_^j+^ and z_i_^j+^ fragments of [P2 + 3H]^3+^ ……... | S6 |
| **Figure S2.** Pseudo-MS^3^ of a_2_^+^ and z_2_^+^ fragments of [P2 + 3H]^3+^ ……………………… | S7 |
| **Figure S3.** MS/MS of [P1 + 3H]^3+^ at *m/z* 676.6 ……………………………………….. | S8 |
| **Figure S4.** MS/MS of [P3 + 3H]^3+^ at *m/z* 695.3 ……………………………………….. | S8 |
| **Figure S5.** MS/MS of [P4 + 3H]^3+^ at *m/z* 770.1 ……………………………………….. | S9 |
| **Figure S6.** MS/MS of [P5 + 3H]^3+^ at *m/z* 788.8 ……………………………………….. | S9 |
| **Figure S7.** MS/MS of [P6 + 3H]^3+^ at *m/z* 751.4 ……………………………………….. | S10 |
| **Figure S8.** MS/MS of [P7 + 3H]^3+^ at *m/z* 751.4 ……………………………………….. | S10 |
| **Table S8.** Accurate mass measurement of a_i_^j+^ and w_i_^j+^ fragments of [P8 + 5H]^5+^ …….. | S11 |
| **Table S9.** Accurate mass measurement of d_i_^j+^ and z_i_^j+^ fragments of [P8 + 5H]^5+^ ……... | S12 |


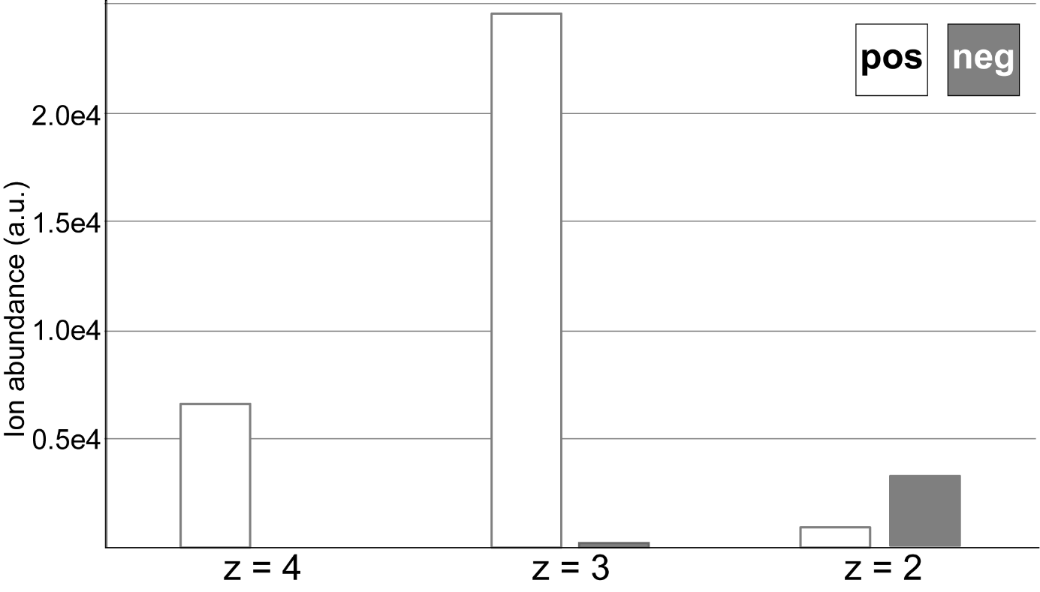


**Figure S1.** Absolute abundance of deprotonated (grey) and protonated (white) P4 molecules as a function of their charge state

| error, ppm | *m/z*_exp_ | *m/z*_th_ | composition | ↓ |  | ↑ | composition | *m/z*_th_ | *m/z*_exp_ | error, ppm |
| --- | --- | --- | --- | --- | --- | --- | --- | --- | --- | --- |
|  |  |  | *n.e.* | a_1_^–^ | **00** | w_8_^2–^ | *n.e.* |  |  |  |
| + 4.9 | 365.2229 | 365.2211 | C_16_H_34_N_2_O_5_P^–^ | a_2_^–^ | **01** | w_7_^2–^ | C_65_H_137_N_9_O_36_P_8_^2–^ | 933.8359 | *n.d.* |  |
| + 2.5 | 602.3356 | 602.3341 | C_25_H_54_N_3_O_9_P_2_^–^ | a_3_^–^ | **01** | w_6_^2–^ | C_56_H_117_N_8_O_32_P_7_^2–^ | 815.2974 | 815.2976 | + 0.2 |
| + 0.5 | 839.4475 | 839.4471 | C_34_H_74_N_4_O_13_P_3_^–^ | a_4_^–^ | **01** | w_5_^–^ | C_47_H_98_N_7_O_28_P_6_^–^ | 1394.4891 | 1394.4847 | – 3.2 |
| – 1.0 | 1048.5277 | 1048.5288 | C_41_H_90_N_5_O_17_P_4_^–^ | a_5_^–^ | **00** | w_4_^–^ | C_38_H_78_N_6_O_24_P_5_^–^ | 1157.3761 | 1157.3742 | – 1.6 |
| – 2.3 | 1257.6076 | 1257.6105 | C_48_H_106_N_6_O_21_P_5_^–^ | a_6_^–^ | **00** | w_3_^–^ | C_31_H_62_N_5_O_20_P_4_^–^ | 948.2944 | 948.2942 | – 0.2 |
| – 2.9 | 1466.6879 | 1466.6922 | C_55_H_122_N_7_O_25_P_6_^–^ | a_7_^–^ | **00** | w_2_^–^ | C_24_H_46_N_4_O_16_P_3_^–^ | 739.2127 | 739.2138 | + 1.5 |
| – 3.4 | 1675.7682 | 1675.7739 | C_62_H_138_N_8_O_29_P_7_^–^ | a_8_^–^ | **00** | w_1_^–^ | C_17_H_30_N_3_O_12_P_2_^–^ | 530.1310 | 530.1331 | + 4.0 |
|  |  |  |  |  | T | w_0_^–^ | C_10_H_14_N_2_O_8_P^–^ | 321.0493 | 321.0520 | + 8.4 |

**Table S1.** Accurate mass measurement of a_i_^j–^ and w_i_^j–^ fragments of [P2 – 2H]^2–^, using the precursor ion (C_72_H_152_N_10_O_37_P_8_^2–^, *m/z* 998.4116) as an internal standard. P2 sequence: α-00-01-01-01-00-00-00-00-ω, with 00: C_7_H_16_NO_4_P (209.0817 Da), 01: C_9_H_20_NO_4_P (237.1130 Da), α: HO (17.0027 Da) and ω: C_10_H_13_N_2_O_4_ (225.0875 Da). *n.e.*: not expected.

| error, ppm | *m/z*_exp_ | *m/z*_th_ | composition | ↓ |  | ↑ | composition | *m/z*_th_ | *m/z*_exp_ | error, ppm |
| --- | --- | --- | --- | --- | --- | --- | --- | --- | --- | --- |
|  | *n.d.* | 146.1187 | C_7_H_16_NO_2_^–^ | b_1_^–^ | **00** | x_8_^2–^ | *n.e.* |  |  |  |
| + 5.5 | 383.2337 | 383.2316 | C_16_H_36_N_2_O_6_P^–^ | b_2_^–^ | **01** | x_7_^2–^ | C_65_H_135_N_9_O_35_P_8_^2–^ | 924.8486 | 924.8481 | – 0.5 |
| + 2.4 | 620.3461 | 620.3446 | C_25_H_56_N_3_O_10_P_2_^–^ | b_3_^–^ | **01** | x_6_^2–^ | C_56_H_115_N_8_O_31_P_7_^2–^ | 806.2921 | 806.2926 | + 0.6 |
| + 0.3 | 857.4579 | 857.4576 | C_34_H_76_N_4_O_14_P_3_^–^ | b_4_^–^ | **01** | x_5_^–^ | C_47_H_96_N_7_O_27_P_6_^–^ | 1376.4785 | 1376.4745 | – 2.9 |
| – 1.1 | 1066.5381 | 1066.5393 | C_41_H_92_N_5_O_18_P_4_^–^ | b_5_^–^ | **00** | x_4_^–^ | C_38_H_76_N_6_O_23_P_5_^–^ | 1139.3655 | 1139.3636 | – 1.7 |
| – 2.3 | 1275.6181 | 1275.6210 | C_48_H_108_N_6_O_22_P_5_^–^ | b_6_^–^ | **00** | x_3_^–^ | C_31_H_60_N_5_O_19_P_4_^–^ | 930.2839 | 930.2834 | – 0.5 |
| – 3.0 | 1484.6982 | 1484.7027 | C_55_H_124_N_7_O_26_P_6_^–^ | b_7_^–^ | **00** | x_2_^–^ | C_24_H_44_N_4_O_15_P_3_^–^ | 721.2022 | 721.2032 | + 1.4 |
| – 2.0 | 846.3869 | 846.3886 | C_62_H_139_N_8_O_30_P_7_^2–^ | b_8_^2–^ | **00** | x_1_^–^ | C_17_H_28_N_3_O_11_P_2_^–^ | 512.1205 | 512.1225 | + 3.9 |
|  |  |  |  |  | T |  |  |  |  |  |

**Table S2.** Accurate mass measurement of b_i_^j–^ and x_i_^j–^ fragments of [P2 – 2H]^2–^, using the precursor ion (C_72_H_152_N_10_O_37_P_8_^2–^, *m/z* 998.4116) as an internal standard. P2 sequence: α-00-01-01-01-00-00-00-00-ω, with 00: C_7_H_16_NO_4_P (209.0817 Da), 01: C_9_H_20_NO_4_P (237.1130 Da), α: HO (17.0027 Da) and ω: C_10_H_13_N_2_O_4_ (225.0875 Da). *n.e.*: not expected. *n.d.*: not detected.

| error, ppm | *m/z*_exp_ | *m/z*_th_ | composition | ↓ |  | ↑ | composition | *m/z*_th_ | *m/z*_exp_ | error, ppm |
| --- | --- | --- | --- | --- | --- | --- | --- | --- | --- | --- |
|  |  |  | *n.e.* | c_1_^–^ | **00** | y_8_^2–^ | *n.e.* |  |  |  |
| + 4.9 | 445.1896 | 445.1874 | C_16_H_35_N_2_O_8_P_2_^–^ | c_2_^–^ | **01** | y_7_^2–^ | C_65_H_136_N_9_O_33_P_7_^2–^ | 893.8707 | 893.8705 | – 0.2 |
| + 2.1 | 682.3018 | 682.3004 | C_25_H_55_N_3_O_12_P_3_^–^ | c_3_^–^ | **01** | y_6_^2–^ | C_56_H_116_N_8_O_29_P_6_^2–^ | 775.3142 | 775.3140 | – 0.3 |
| – 0.1 | 919.4133 | 919.4134 | C_34_H_75_N_4_O_16_P_4_^–^ | c_4_^–^ | **01** | y_5_^–^ | C_47_H_97_N_7_O_25_P_5_^–^ | 1314.5228 | 1314.5198 | – 2.3 |
| – 1.4 | 1128.4935 | 1128.4951 | C_41_H_91_N_5_O_20_P_5_^–^ | c_5_^–^ | **00** | y_4_^–^ | C_38_H_77_N_6_O_21_P_4_^–^ | 1077.4098 | 1077.4086 | – 1.1 |
| – 2.7 | 1337.5732 | 1337.5768 | C_48_H_107_N_6_O_24_P_6_^–^ | c_6_^–^ | **00** | y_3_^–^ | C_31_H_61_N_5_O_17_P_3_^–^ | 868.3281 | 868.3284 | + 0.3 |
| – 3.0 | 1546.6538 | 1546.6585 | C_55_H_123_N_7_O_28_P_7_^–^ | c_7_^–^ | **00** | y_2_^–^ | C_24_H_45_N_4_O_13_P_2_^–^ | 659.2464 | 659.2478 | + 2.1 |
| – 0.3 | 877.3662 | 877.3665 | C_62_H_138_N_8_O_32_P_8_^2–^ | c_8_^2–^ | **00** | y_1_^–^ | C_17_H_29_N_3_O_9_P_1_^–^ | 450.1647 | 450.1670 | + 5.1 |
|  |  |  |  |  | T |  |  |  |  |  |

**Table S3.** Accurate mass measurement of c_i_^j–^ and y_i_^j–^ fragments of [P2 – 2H]^2–^, using the precursor ion (C_72_H_152_N_10_O_37_P_8_^2–^, *m/z* 998.4116) as an internal standard. P2 sequence: α-00-01-01-01-00-00-00-00-ω, with 00: C_7_H_16_NO_4_P (209.0817 Da), 01: C_9_H_20_NO_4_P (237.1130 Da), α: HO (17.0027 Da) and ω: C_10_H_13_N_2_O_4_ (225.0875 Da). *n.e.*: not expected.

| error, ppm | *m/z*_exp_ | *m/z*_th_ | composition | ↓ |  | ↑ | composition | *m/z*_th_ | *m/z*_exp_ | error, ppm |
| --- | --- | --- | --- | --- | --- | --- | --- | --- | --- | --- |
| + 6.6 | 226.0865 | 226.0850 | C_7_H_17_NO_5_P^–^ | d_1_^–^ | **00** | z_8_^2–^ | C_72_H_150_N_10_O_36_P_8_^2–^ | 989.4063 | 989.4023 | – 4.0 |
| + 4.7 | 463.2002 | 463.1980 | C_16_H_37_N_2_O_9_P_2_^–^ | d_2_^–^ | **01** | z_7_^2–^ | C_65_H_134_N_9_O_32_P_7_^2–^ | 884.8655 | 884.8652 | – 0.3 |
| + 1.7 | 700.3122 | 700.3110 | C_25_H_57_N_3_O_13_P_3_^–^ | d_3_^–^ | **01** | z_6_^–^ | C_56_H_115_N_8_O_28_P_6_^–^ | 1533.6252 | 1533.6204 | – 3.1 |
| – 0.3 | 937.4237 | 937.4240 | C_34_H_77_N_4_O_17_P_4_^–^ | d_4_^–^ | **01** | z_5_^–^ | C_47_H_95_N_7_O_24_P_5_^–^ | 1296.5122 | 1296.5091 | – 2.4 |
| – 1.6 | 1146.5039 | 1146.5057 | C_41_H_93_N_5_O_21_P_5_^–^ | d_5_^–^ | **00** | z_4_^–^ | C_38_H_75_N_6_O_20_P_4_^–^ | 1059.3992 | 1059.3982 | – 0.9 |
| – 2.4 | 1355.5841 | 1355.5874 | C_48_H_109_N_6_O_25_P_6_^–^ | d_6_^–^ | **00** | z_3_^–^ | C_31_H_59_N_5_O_16_P_3_^–^ | 850.3175 | 850.3179 | + 0.5 |
| + 0.6 | 781.8314 | 781.8309 | C_55_H_124_N_7_O_29_P_7_^2–^ | d_7_^2–^ | **00** | z_2_^–^ | C_24_H_43_N_4_O_12_P_2_^–^ | 641.2358 | 641.2373 | + 2.3 |
| + 3.7 | 886.3750 | 886.3717 | C_62_H_140_N_8_O_33_P_8_^2–^ | d_8_^2–^ | **00** | z_1_^–^ | C_17_H_27_N_3_O_8_P_1_^–^ | 432.1541 | 432.1560 | + 4.4 |
|  |  |  |  |  | T |  |  |  |  |  |

**Table S4.** Accurate mass measurement of d_i_^j–^ and z_i_^j–^ fragments of [P2 – 2H]^2–^, using the precursor ion (C_72_H_152_N_10_O_37_P_8_^2–^, *m/z* 998.4116) as an internal standard. P2 sequence: α-00-01-01-01-00-00-00-00-ω, with 00: C_7_H_16_NO_4_P (209.0817 Da), 01: C_9_H_20_NO_4_P (237.1130 Da), α: HO (17.0027 Da) and ω: C_10_H_13_N_2_O_4_ (225.0875 Da).

| 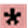 | 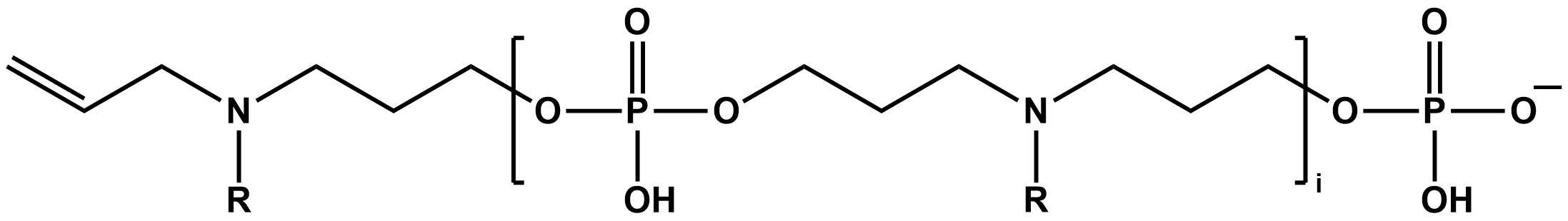 | | | | | |
| --- | --- | --- | --- | --- | --- | --- |
|  | [00]_0_ | [00]_1_ | [00]_2_ | [00]_3_ | [00]_4_ | [00]_5_ |
| [01]_0_ | - | 208.1^–^ | 417.2^1–^ | 626.3^1–^ | 835.4^1–^ | - |
| [01]_1_ | 236.1^1–^ | **445.2^1–^** | 654.3^1–^ | 863.4^1–^ | 1072.4^1–^ | - |
| [01]_2_ | 473.2^1–^ | **682.3^1–^** | 891.4^1–^ | 1100.5^1–^ | 1309.6^1–^ | - |
| [01]_3_ | 710.4^1–^ | **919.4^1–^** | **1128.6^1–^** | **1337.6^1–^** | **1546.8^1–^** | **877.4^2–^** |

**Table S5.** Structure and *m/z* values of internal fragments with *m/z* = ∑m(Mi) – 1, as a function of their co-monomeric composition. Some of these internal fragments share the same elemental composition and thus the same *m/z* value (in bold) as c_i_^j–^ fragments generated during CID of [P2 – 2H]^2–^ (Figure 2).

| error, ppm | *m/z*_exp_ | *m/z*_th_ | composition | ↓ |  | ↑ | composition | *m/z*_th_ | *m/z*_exp_ | error, ppm |
| --- | --- | --- | --- | --- | --- | --- | --- | --- | --- | --- |
| – 7.7 | 130.1216 | 130.1226 | C_7_H_16_NO^+^ | a_1_^+^ | **00** | w_8_^2+^ | *n.e.* |  |  |  |
| – 1.4 | 367.2351 | 367.2356 | C_16_H_36_N_2_O_5_P^+^ | a_2_^+^ | **01** | w_7_^2+^ | C_65_H_141_N_9_O_36_P_8_^2+^ | 935.8685 | 935.8686 | + 0.1 |
| – 3.6 | 302.6769 | 302.6780 | C_25_H_57_N_3_O_9_P_2_^2+^ | a_3_^2+^ | **01** | w_6_^2+^ | C_56_H_121_N_8_O_32_P_7_^2+^ | 817.3120 | 817.3120 | 0 |
| – 2.1 | 421.2336 | 421.2345 | C_34_H_77_N_4_O_13_P_3_^2+^ | a_4_^2+^ | **01** | w_5_^2+^ | C_47_H_101_N_7_O_28_P_6_^2+^ | 698.7555 | 698.7555 | 0 |
| – 0.8 | 525.7749 | 525.7753 | C_41_H_93_N_5_O_17_P_4_^2+^ | a_5_^2+^ | **00** | w_4_^+^ | C_38_H_80_N_6_O_24_P_5_^+^ | 1159.3907 | 1159.3923 | + 1.4 |
| – 0.5 | 630.3159 | 630.3162 | C_48_H_109_N_6_O_21_P_5_^2+^ | a_6_^2+^ | **00** | w_3_^+^ | C_31_H_64_N_5_O_20_P_4_^+^ | 950.3090 | 950.3097 | + 0.7 |
| + 0.3 | 734.8572 | 734.8570 | C_55_H_125_N_7_O_25_P_6_^2+^ | a_7_^2+^ | **00** | w_2_^+^ | C_24_H_48_N_4_O_16_P_3_^+^ | 741.2273 | 741.2283 | + 1.3 |
| – 1.4 | 559.9335 | 559.9343 | C_62_H_142_N_8_O_29_P_7_^3+^ | a_8_^3+^ | **00** | w_1_^+^ | C_17_H_32_N_3_O_12_P_2_^+^ | 532.1456 | 532.1458 | + 0.4 |
|  |  |  |  |  | T |  |  |  |  |  |

**Table S6.** Accurate mass measurement of a_i_^j+^ and w_i_^j+^ fragments of [P2 + 3H]^3+^, using the precursor ion (C_72_H_157_N_10_O_37_P_8_^3+^, *m/z* 667.2865) as an internal standard. P2 sequence: α-00-01-01-01-00-00-00-00-ω, with 00: C_7_H_16_NO_4_P (209.0817 Da), 01: C_9_H_20_NO_4_P (237.1130 Da), α: HO (17.0027 Da) and ω: C_10_H_13_N_2_O_4_ (225.0875 Da). *n.e.*: not expected.

| error, ppm | *m/z*_exp_ | *m/z*_th_ | composition | ↓ |  | ↑ | composition | *m/z*_th_ | *m/z*_exp_ | error, ppm |
| --- | --- | --- | --- | --- | --- | --- | --- | --- | --- | --- |
| – 3.5 | 228.0987 | 228.0995 | C_7_H_19_NO_5_P^+^ | d_1_^+^ | **00** | z_8_^3+^ | *n.e.* |  |  |  |
| – 0.2 | 465.2124 | 465.2125 | C_16_H_39_N_2_O_9_P_2_^+^ | d_2_^+^ | **01** | z_7_^3+^ | C_65_H_139_N_9_O_32_P_7_^3+^ | 591.5891 | 591.5882 | – 1.5 |
| – 0.9 | 702.3261 | 702.3255 | C_25_H_59_N_3_O_13_P_3_^+^ | d_3_^+^ | **01** | z_6_^2+^ | C_56_H_118_N_8_O_28_P_6_^2+^ | 768.3235 | 768.3235 | 0 |
| – 1.9 | 470.2220 | 470.2229 | C_34_H_80_N_4_O_17_P_4_^2+^ | d_4_^2+^ | **01** | z_5_^2+^ | C_47_H_98_N_7_O_24_P_5_^2+^ | 649.7670 | 649.7668 | – 0.3 |
| – 0.7 | 574.7634 | 574.7638 | C_41_H_96_N_5_O_21_P_5_^2+^ | d_5_^2+^ | **00** | z_4_^2+^ | C_38_H_78_N_6_O_20_P_4_^2+^ | 531.2105 | 531.2103 | – 0.4 |
| – 0.3 | 679.3044 | 679.3046 | C_48_H_112_N_6_O_25_P_6_^2+^ | d_6_^2+^ | **00** | z_3_^+^ | C_31_H_61_N_5_O_16_P_3_^+^ | 852.3321 | 852.3332 | + 1.3 |
| + 0.4 | 783.8457 | 783.8454 | C_55_H_128_N_7_O_29_P_7_^2+^ | d_7_^2+^ | **00** | z_2_^+^ | C_24_H_45_N_4_O_12_P_2_^+^ | 643.2504 | 643.2510 | + 0.9 |
| – 2.4 | 592.5919 | 592.5933 | C_62_H_145_N_8_O_33_P_8_^3+^ | d_8_^3+^ | **00** | z_1_^+^ | C_17_H_29_N_3_O_8_P^+^ | 434.1687 | 434.1684 | – 0.7 |
|  |  |  |  |  | T |  |  |  |  |  |

**Table S7.** Accurate mass measurement of d_i_^j+^ and z_i_^j+^ fragments of [P2 + 3H]^3+^, using the precursor ion (C_72_H_157_N_10_O_37_P_8_^3+^, *m/z* 667.2865) as an internal standard. P2 sequence: α-00-01-01-01-00-00-00-00-ω, with 00: C_7_H_16_NO_4_P (209.0817 Da), 01: C_9_H_20_NO_4_P (237.1130 Da), α: HO (17.0027 Da) and ω: C_10_H_13_N_2_O_4_ (225.0875 Da). *n.e.*: not expected.

**
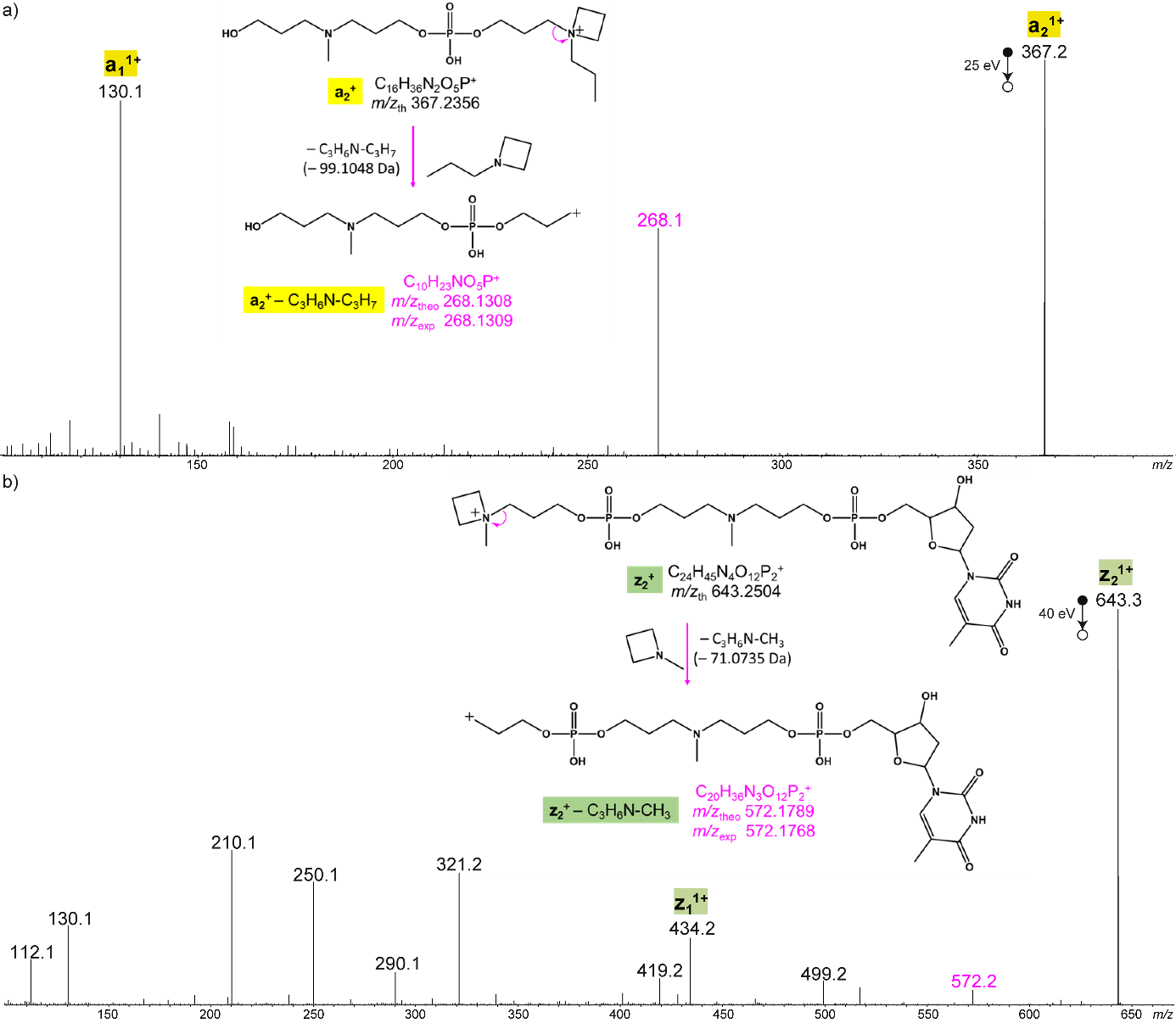
**

**Figure S2.** Pseudo-MS^3^ experiments performed by raising the cone voltage from +75 V to +200 V to induce in-source fragmentation of [P2 + 3H]^3+^ and enable subsequent mass-selection of fragments for further CID as shown for a) a_2_^+^ at *m/z* 367.2 and b) z_2_^+^ at *m/z* 643.3. From both ions, elimination of the nitrogen-containing four-membered ring is shown in pink. For a_2_^+^ of sequence α-00-01, the R group connected to the charged nitrogen is C_3_H_8_ so the mass of released four-membered ring is 99.1 Da. For z_2_^+^ of sequence ω -00-00, the R group connected to the charged nitrogen is CH_3_ so the mass of released four-membered ring is 77.1 Da.


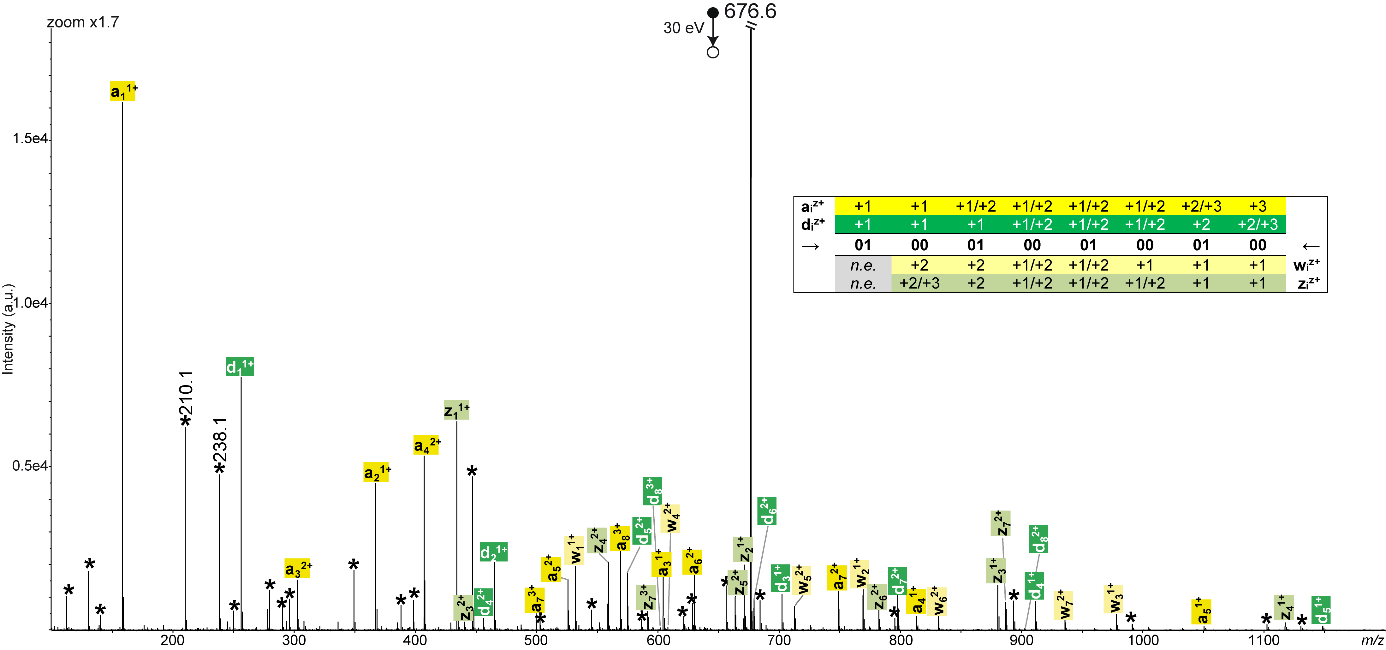


**Figure S3.** MS/MS of [P1 + 3H]^3+^ at *m/z* 676.6. Coverage of the 01-00-01-00-01-00-01-00 sequence is shown in the inset table which indicates the charge state(s) of detected fragments (*n.e.*: not expected). Asterisks designate secondary fragments, including protonated monomers [00 + H]^+^ at *m/z* 210.1 and [01 + H]^+^ at *m/z* 238.1.


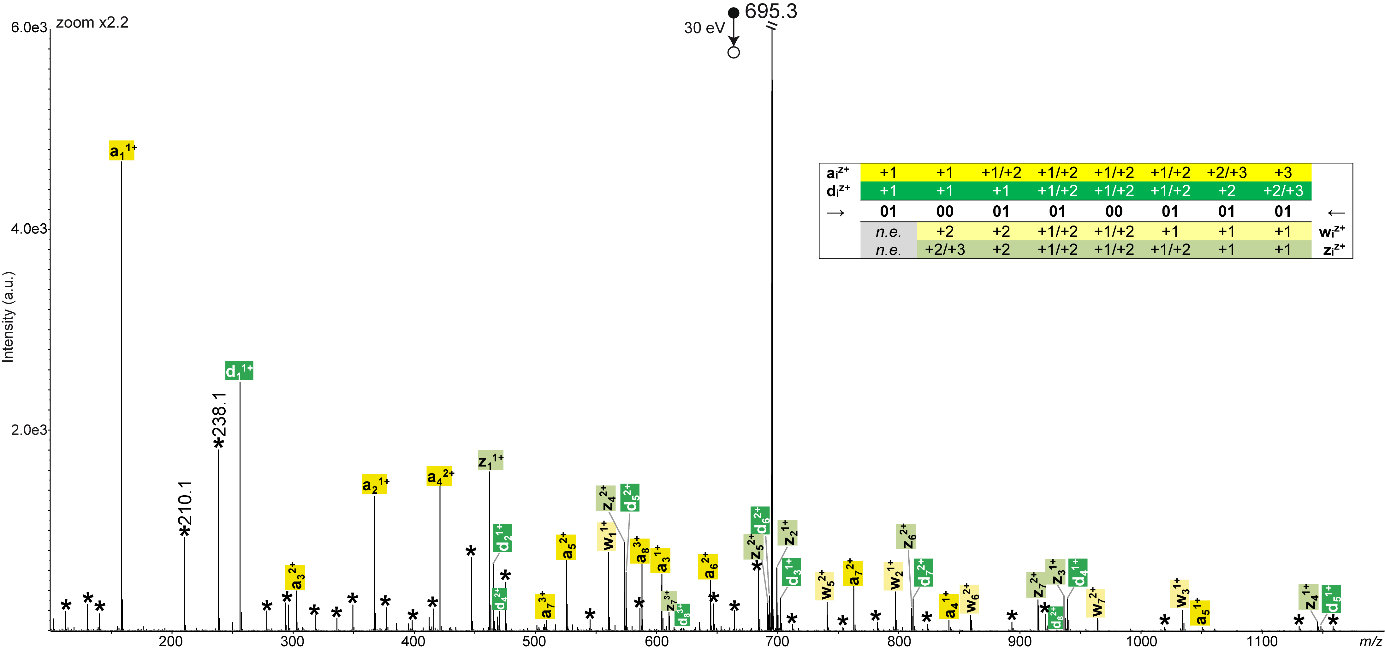


**Figure S4.** MS/MS of [P3 + 3H]^3+^ at *m/z* 695.3. Coverage of the 01-00-01-01-00-01-01-01 sequence is shown in the inset table which indicates the charge state(s) of detected fragments (*n.e.*: not expected). Asterisks designate secondary fragments, including protonated monomers [00 + H]^+^ at *m/z* 210.1 and [01 + H]^+^ at *m/z* 238.1.


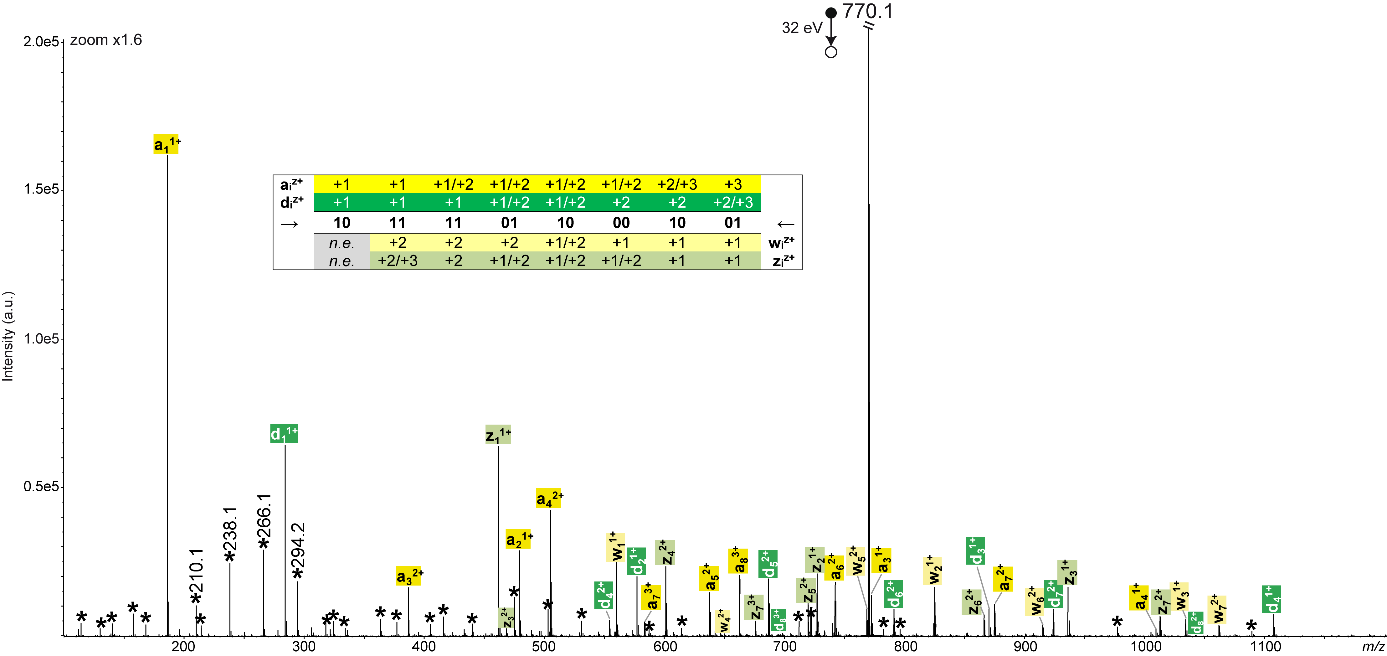


**Figure S5.** MS/MS of [P4 + 3H]^3+^ at *m/z* 770.1. Coverage of the 10-11-11-01-10-00-10-01 sequence is shown in the inset table which indicates the charge state(s) of detected fragments (*n.e.*: not expected). Asterisks designate secondary fragments, including protonated monomers [00 + H]^+^ at *m/z* 210.1, [01 + H]^+^ at *m/z* 238.1, [10 + H]^+^ at *m/z* 266.1 and [11 + H]^+^ at *m/z* 294.2.


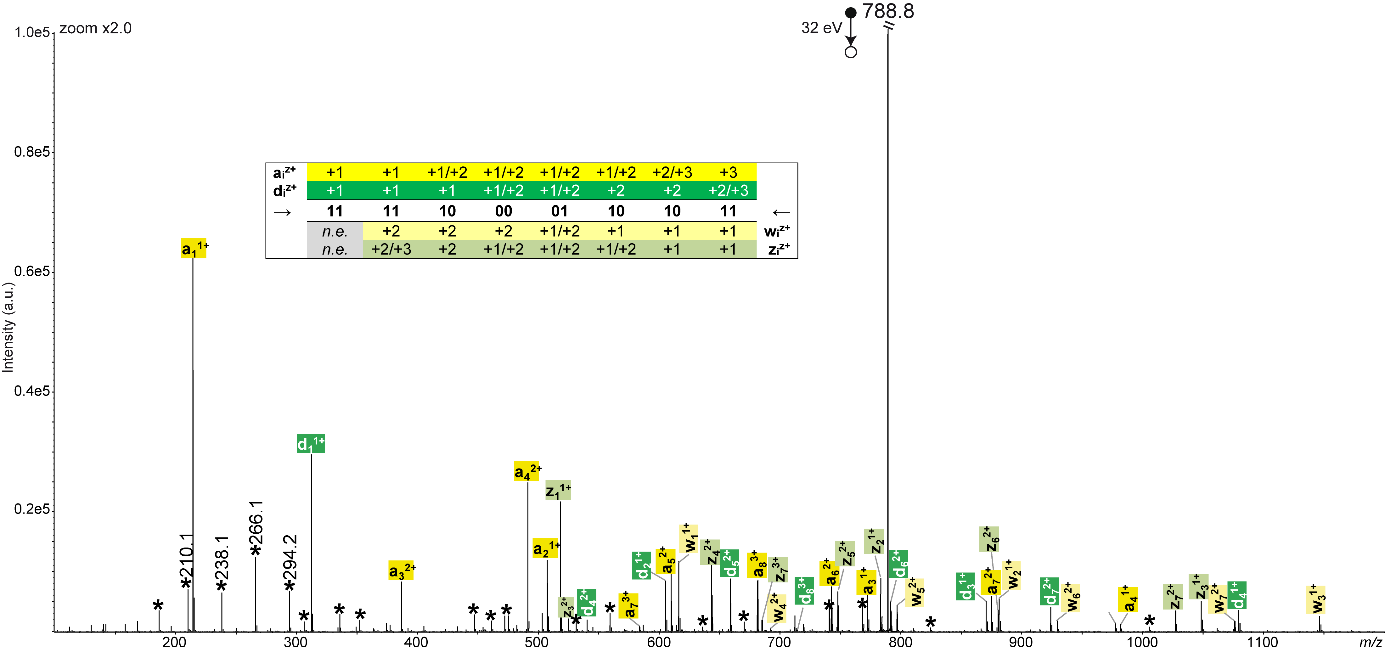


**Figure S6.** MS/MS of [P5 + 3H]^3+^ at *m/z* 788.8. Coverage of the 11-11-10-00-01-10-10-11 sequence is shown in the inset table which indicates the charge state(s) of detected fragments (*n.e.*: not expected). Asterisks designate secondary fragments, including protonated monomers [00 + H]^+^ at *m/z* 210.1, [01 + H]^+^ at *m/z* 238.1, [10 + H]^+^ at *m/z* 266.1 and [11 + H]^+^ at *m/z* 294.2.


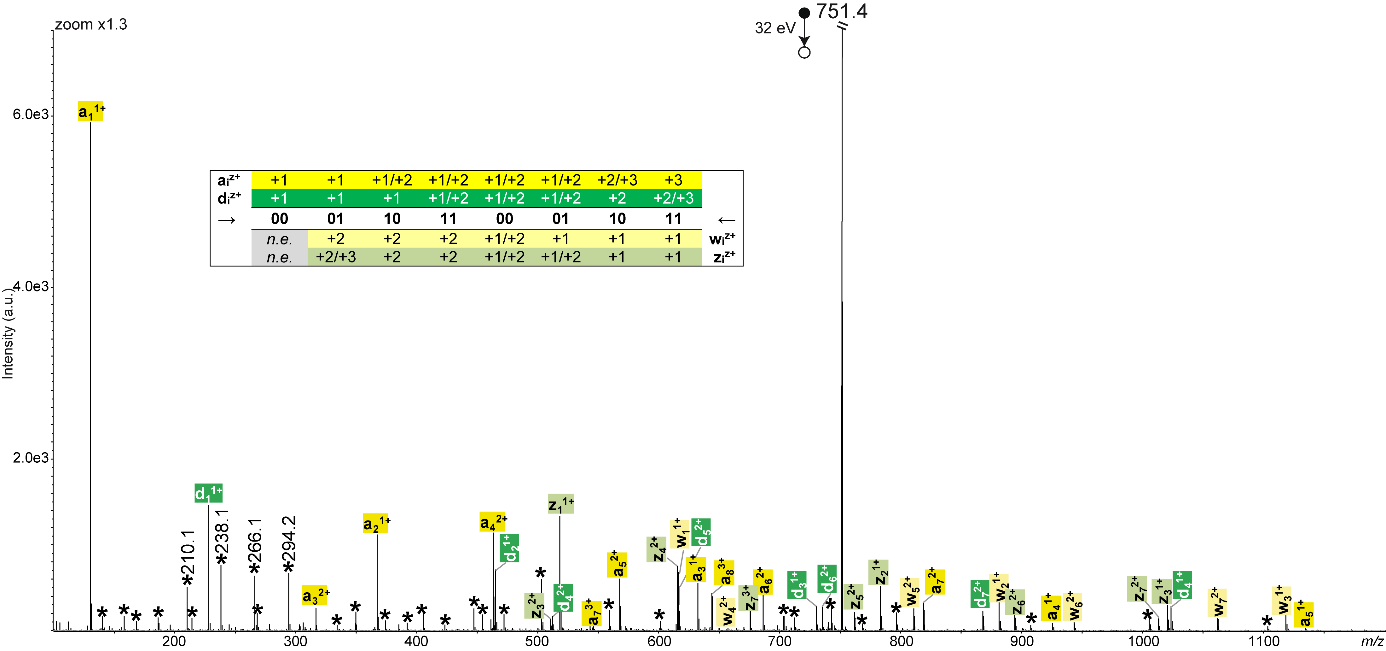


**Figure S7.** MS/MS of [P6 + 3H]^3+^ at *m/z* 751.4. Coverage of the 00-01-10-11-00-01-10-11 sequence is shown in the inset table which indicates the charge state(s) of detected fragments (*n.e.*: not expected). Asterisks designate secondary fragments, including protonated monomers [00 + H]^+^ at *m/z* 210.1, [01 + H]^+^ at *m/z* 238.1, [10 + H]^+^ at *m/z* 266.1 and [11 + H]^+^ at *m/z* 294.2.


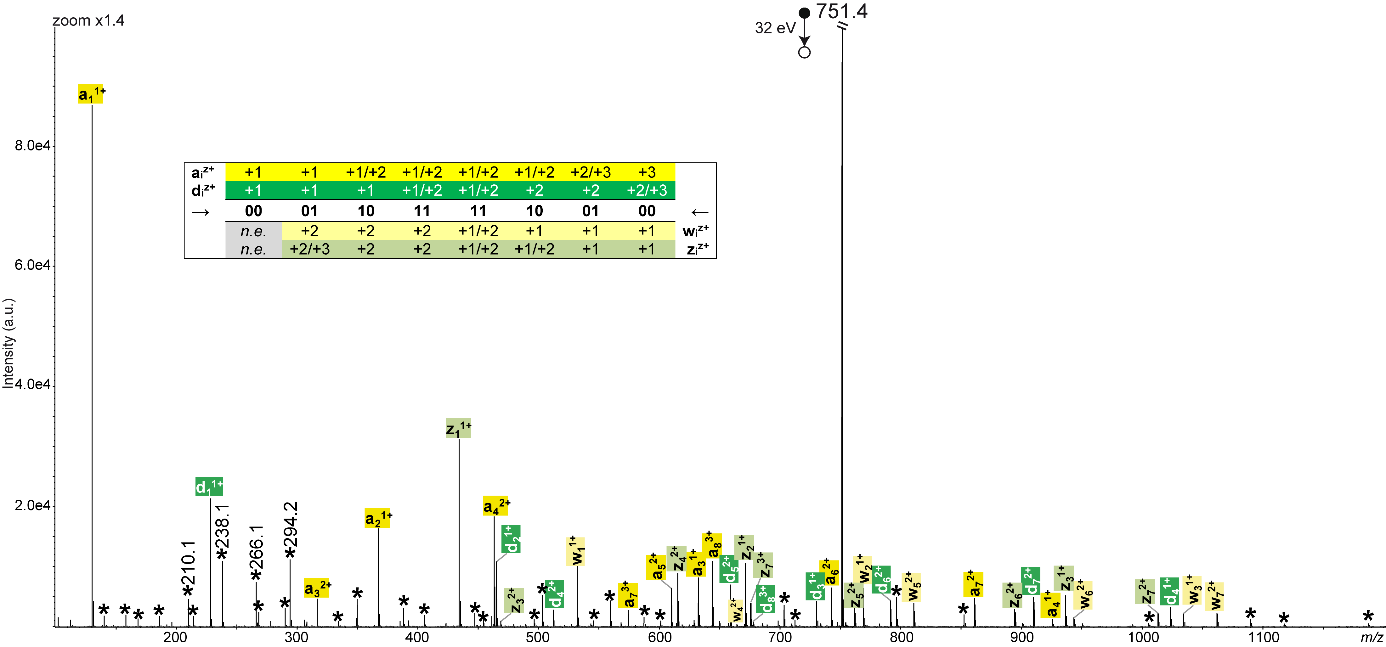


**Figure S8.** MS/MS of [P7 + 3H]^3+^ at *m/z* 751.4. Coverage of the 00-01-10-11-11-10-01-00 sequence is shown in the inset table which indicates the charge state(s) of detected fragments (*n.e.*: not expected). Asterisks designate secondary fragments, including protonated monomers [00 + H]^+^ at *m/z* 210.1, [01 + H]^+^ at *m/z* 238.1, [10 + H]^+^ at *m/z* 266.1 and [11 + H]^+^ at *m/z* 294.2.

| error  (ppm) | *m/z*_exp_ | *m/z*_th_ | composition | ↓ |  | ↑ | composition | *m/z*_th_ | *m/z*_exp_ | error  (ppm) |
| --- | --- | --- | --- | --- | --- | --- | --- | --- | --- | --- |
| – 0.8 | 130.1225 | 130.1226 | C_7_H_16_NO^+^ | a_1_^+^ | **00** | w_16_^4+^ | *n.e.* |  |  |  |
| – 0.8 | 367.2353 | 367.2356 | C_16_H_36_N_2_O_5_P^+^ | a_2_^+^ | **01** | w_15_^4+^ | C_131_H_291_N_17_O_68_P_16_^4+^ | 921.6404 | 921.6403 | – 0.1 |
| – 0.8 | 604.3481 | 604.3486 | C_25_H_56_N_3_O_9_P_2_^+^ | a_3_^+^ | **01** | w_14_^4+^ | C_122_H_271_N_16_O_64_P_15_^4+^ | 862.3622 | 862.3617 | – 0.6 |
| + 0.2 | 421.2346 | 421.2345 | C_34_H_77_N_4_O_13_P_3_^2+^ | a_4_^2+^ | **01** | w_13_^4+^ | C_113_H_251_N_15_O_60_P_14_^4+^ | 803.0839 | 803.0841 | + 0.2 |
| + 0.2 | 539.7911 | 539.7910 | C_43_H_97_N_5_O_17_P_4_^2+^ | a_5_^2+^ | **01** | w_12_^4+^ | C_104_H_231_N_14_O_56_P_13_^4+^ | 743.8057 | 743.8062 | + 0.7 |
| + 0.2 | 644.3319 | 644.3318 | C_50_H_113_N_6_O_21_P_5_^2+^ | a_6_^2+^ | **00** | w_11_^3+^ | C_95_H_210_N_13_O_52_P_12_^3+^ | 912.3675 | 912.3674 | – 0.1 |
| + 0.1 | 748.8727 | 748.8726 | C_57_H_129_N_7_O_25_P_6_^2+^ | a_7_^2+^ | **00** | w_10_^3+^ | C_88_H_194_N_12_O_48_P_11_^3+^ | 842.6736 | 842.6736 | 0 |
| + 0.9 | 569.2786 | 569.2781 | C_64_H_146_N_8_O_29_P_7_^3+^ | a_8_^3+^ | **00** | w_9_^3+^ | C_81_H_178_N_11_O_44_P_10_^3+^ | 772.9796 | 772.9804 | + 1.0 |
| + 0.6 | 638.9724 | 638.9720 | C_71_H_162_N_9_O_33_P_8_^3+^ | a_9_^3+^ | **00** | w_8_^2+^ | C_74_H_161_N_10_O_40_P_9_^2+^ | 1054.4250 | 1054.4240 | – 0.9 |
| + 0.6 | 708.6663 | 708.6659 | C_78_H_178_N_10_O_37_P_9_^3+^ | a_10_^3+^ | **00** | w_7_^2+^ | C_67_H_145_N_9_O_36_P_8_^2+^ | 949.8841 | 949.8834 | – 0.7 |
| + 1.0 | 584.2731 | 584.2725 | C_85_H_195_N_11_O_41_P_10_^4+^ | a_11_^4+^ | **00** | w_6_^2+^ | C_60_H_129_N_8_O_32_P_7_^2+^ | 845.3433 | 845.3429 | – 0.5 |
| – 0.9 | 643.2993 | 643.2999 | C_94_H_215_N_12_O_45_P_11_^4+^ | a_12_^4+^ | **01** | w_5_^2+^ | C_53_H_113_N_7_O_28_P_6_^2+^ | 740.8024 | 740.8026 | + 0.3 |
| – 0.3 | 702.5780 | 702.5782 | C_103_H_235_N_13_O_49_P_12_^4+^ | a_13_^4+^ | **01** | w_4_^+^ | C_44_H_92_N_6_O_24_P_5_^+^ | 1243.4846 | 1243.4843 | – 0.2 |
| – 0.5 | 761.8560 | 761.8564 | C_112_H_255_N_14_O_53_P_13_^4+^ | a_14_^4+^ | **01** | w_3_^+^ | C_35_H_72_N_5_O_20_P_4_^+^ | 1006.3716 | 1006.3702 | – 1.4 |
| – 1.2 | 821.1337 | 821.1347 | C_121_H_275_N_15_O_57_P_14_^4+^ | a_15_^4+^ | **01** | w_2_^+^ | C_26_H_52_N_4_O_16_P_3_^+^ | 769.2586 | 769.2578 | – 1.0 |
| + 0.4 | 698.9258 | 698.9255 | C_128_H_292_N_16_O_61_P_15_^5+^ | a_16_^5+^ | **00** | w_1_^+^ | C_17_H_32_N_3_O_12_P_2_^+^ | 532.1456 | 532.1454 | – 0.4 |
|  |  |  |  |  | T |  |  |  |  |  |

**Table S8.** Accurate mass measurement of a_i_^j+^ and w_i_^j+^ fragments of [P8 + 5H]^5+^, using the precursor ion (C_138_H_307_N_18_O_69_P_6_^5+^, *m/z* 763.3368) as an internal standard. P8 sequence: α-00-01-01-01-01-00-00-00-00-00-00-01-01-01-01-00-ω, with 00: C_7_H_16_NO_4_P (209.0817 Da), 01: C_9_H_20_NO_4_P (237.1130 Da), α: HO (17.0027 Da) and ω: C_10_H_13_N_2_O_4_ (225.0875 Da). *n.e.*: not expected.

| error  (ppm) | *m/z*_exp_ | *m/z*_th_ | composition | ↓ |  | ↑ | composition | *m/z*_th_ | *m/z*_exp_ | error  (ppm) |
| --- | --- | --- | --- | --- | --- | --- | --- | --- | --- | --- |
| – 0.4 | 228.0994 | 228.0995 | C_7_H_19_NO_5_P^+^ | d_1_^+^ | **00** | z_16_^4+^ | *n.e.* |  |  |  |
| – 0.6 | 465.2122 | 465.2125 | C_16_H_39_N_2_O_9_P_2_^+^ | d_2_^+^ | **01** | z_15_^4+^ | C_131_H_288_N_17_O_64_P_15_^4+^ | 897.1462 | 897.1455 | – 0.8 |
| – 0.9 | 702.3249 | 702.3255 | C_25_H_59_N_3_O_13_P_3_^+^ | d_3_^+^ | **01** | z_14_^4+^ | C_122_H_268_N_16_O_60_P_14_^4+^ | 837.8680 | 837.8671 | – 1.1 |
| – 1.2 | 939.4374 | 939.4385 | C_34_H_79_N_4_O_17_P_4_^+^ | d_4_^+^ | **01** | z_13_^4+^ | C_113_H_248_N_15_O_56_P_13_^4+^ | 778.5897 | 778.5894 | – 0.4 |
| + 0.3 | 588.7796 | 588.7794 | C_43_H_100_N_5_O_21_P_5_^2+^ | d_5_^2+^ | **01** | z_12_^4+^ | C_104_H_228_N_14_O_52_P_12_^4+^ | 719.3115 | 719.3111 | – 0.6 |
| – 0.1 | 693.3201 | 693.3202 | C_50_H_116_N_6_O_25_P_6_^2+^ | d_6_^2+^ | **00** | z_11_^3+^ | C_95_H_207_N_13_O_48_P_11_^3+^ | 879.7085 | 879.7084 | – 0.1 |
| – 0.1 | 797.8610 | 797.8611 | C_57_H_132_N_7_O_29_P_7_^2+^ | d_7_^2+^ | **00** | z_10_^3+^ | C_88_H_191_N_12_O_44_P_10_^3+^ | 810.0146 | 810.0151 | + 0.6 |
| – 0.7 | 902.4013 | 902.4019 | C_64_H_148_N_8_O_33_P_8_^2+^ | d_8_^2+^ | **00** | z_9_^3+^ | C_81_H_175_N_11_O_40_P_9_^3+^ | 740.3207 | 740.3212 | + 0.7 |
| – 0.6 | 1006.9422 | 1006.9428 | C_71_H_164_N_9_O_37_P_9_^2+^ | d_9_^2+^ | **00** | z_8_^3+^ | C_74_H_159_N_10_O_36_P_8_^3+^ | 670.6268 | 670.6272 | + 0.6 |
| – 1.9 | 741.3235 | 741.3249 | C_78_H_181_N_10_O_41_P_10_^3+^ | d_10_^3+^ | **00** | z_7_^2+^ | C_67_H_142_N_9_O_32_P_7_^2+^ | 900.8957 | 900.8948 | – 1.0 |
| + 0.7 | 811.0194 | 811.0188 | C_85_H_197_N_11_O_45_P_11_^3+^ | d_11_^3+^ | **00** | z_6_^2+^ | C_60_H_126_N_8_O_28_P_6_^2+^ | 796.3549 | 796.3550 | + 0.1 |
| + 0.7 | 890.0570 | 890.0564 | C_94_H_217_N_12_O_49_P_12_^3+^ | d_12_^3+^ | **01** | z_5_^2+^ | C_53_H_110_N_7_O_24_P_5_^2+^ | 691.8140 | 691.8131 | – 1.3 |
| – 0.5 | 969.0936 | 969.0941 | C_103_H_237_N_13_O_53_P_13_^3+^ | d_13_^3+^ | **01** | z_4_^2+^ | C_44_H_90_N_6_O_20_P_4_^2+^ | 573.2575 | 573.2569 | – 1.0 |
| + 0.3 | 786.3509 | 786.3507 | C_112_H_258_N_14_O_57_P_14_^4+^ | d_14_^4+^ | **01** | z_3_^+^ | C_35_H_69_N_5_O_16_P_3_^+^ | 908.3947 | 908.3929 | – 2.0 |
| + 0.5 | 845.6293 | 845.6289 | C_121_H_278_N_15_O_61_P_15_^4+^ | d_15_^4+^ | **01** | z_2_^+^ | C_26_H_49_N_4_O_12_P_2_^+^ | 671.2817 | 671.2827 | + 1.5 |
| – 1.3 | 718.5200 | 718.5209 | C_128_H_295_N_16_O_65_P_16_^4+^ | d_16_^5+^ | **00** | z_1_^+^ | C_17_H_29_N_3_O_8_P^+^ | 434.1687 | 434.1684 | – 0.7 |
|  |  |  |  |  | T |  |  |  |  |  |

**Table S9.** Accurate mass measurement of d_i_^j+^ and z_i_^j+^ fragments of [P8 + 5H]^5+^, using the precursor ion (C_138_H_307_N_18_O_69_P_6_^5+^, *m/z* 763.3368) as an internal standard. P8 sequence: α-00-01-01-01-01-00-00-00-00-00-00-01-01-01-01-00-ω, with 00: C_7_H_16_NO_4_P (209.0817 Da), 01: C_9_H_20_NO_4_P (237.1130 Da), α: HO (17.0027 Da) and ω: C_10_H_13_N_2_O_4_ (225.0875 Da). *n.e.*: not expected.
